# Genome-wide analysis of the *WRKY* gene family in the cucumber genome and transcriptome-wide identification of WRKY transcription factors that respond to biotic and abiotic stresses

**DOI:** 10.1186/s12870-020-02625-8

**Published:** 2020-09-25

**Authors:** Chunhua Chen, Xueqian Chen, Jing Han, Wenli Lu, Zhonghai Ren

**Affiliations:** 1 State Key Laboratory of Crop Biology, Shandong Collaborative Innovation Center of Fruit & Vegetable Quality and Efficient Production, Tai’an, People’s Republic of China; 2 Key Laboratory of Biology and Genetic Improvement of Horticultural Crops in Huang-Huai Region, Ministry of Agriculture, College of Horticulture Science and Engineering, Shandong Agricultural University, Tai’an, Shandong 271018 People’s Republic of China

**Keywords:** Cucumber, WRKY, Abiotic stress, Biotic stress, Expression profiling

## Abstract

**Background:**

Cucumber (*Cucumis sativus* L.) is an economically important vegetable crop species. However, it is susceptible to various abiotic and biotic stresses. WRKY transcription factors play important roles in plant growth and development, particularly in the plant response to biotic and abiotic stresses. However, little is known about the expression pattern of *WRKY* genes under different stresses in cucumber.

**Results:**

In the present study, an analysis of the new assembly of the cucumber genome (v3.0) allowed the identification of 61 cucumber *WRKY* genes. Phylogenetic and synteny analyses were performed using related species to investigate the evolution of the cucumber *WRKY* genes. The 61 *CsWRKYs* were classified into three main groups, within which the gene structure and motif compositions were conserved. Tissue expression profiles of the *WRKY* genes demonstrated that 24 *CsWRKY* genes showed constitutive expression (FPKM > 1 in all samples), and some *WRKY* genes showed organ-specific expression, suggesting that these *WRKYs* might be important for plant growth and organ development in cucumber. Importantly, analysis of the *CsWRKY* gene expression patterns revealed that five *CsWRKY* genes strongly responded to both salt and heat stresses, 12 genes were observed to be expressed in response to infection from downy mildew and powdery mildew, and three *CsWRKY* genes simultaneously responded to all treatments analysed. Some *CsWRKY* genes were observed to be induced/repressed at different times after abiotic or biotic stress treatment, demonstrating that cucumber *WRKY* genes might play different roles during different stress responses and that their expression patterns vary in response to stresses.

**Conclusions:**

Sixty-one *WRKY* genes were identified in cucumber, and insight into their classification, evolution, and expression patterns was gained in this study. Responses to different abiotic and biotic stresses in cucumber were also investigated. Our results provide a better understanding of the function of *CsWRKY* genes in improving abiotic and biotic stress resistance in cucumber.

## Background

Throughout their lifecycle, plants frequently encounter many different types of stresses that severely prevent them from reaching optimal growth and that may have a great impact on yield production [1–4]. Some of these stresses are abiotic stress factors such as temperature, drought, salt, and heavy metal stress. By contrast, biotic stress factors involve the interaction of plants with insect, nematode, viral, bacterial, fungal, or oomycete origins that use the plant as a food source [2, 5]. To withstand or cope with these different stresses, plants have evolved a series of adjustment mechanisms including a broad regulation of numerous genes to mediate plant physiological and biochemical processes [6, 7]. Therefore, the study of genes involved in these mechanisms is important for the development of biotechnological tools to enhance desirable agronomic traits, such as plant growth and productivity. Transcription factors (TFs), including members of the AP2/ERF, NAC, MYB, and WRKY families, participate in plant tolerance against abiotic and biotic stresses by modulating the expression of defence-related genes [8–14].

The *WRKY* gene family is one of the largest and most extensively studied TF families in higher plants [15]. Since the first *WRKY* gene was cloned in sweet potato, the identification of *WRKY* genes has been performed in various plant species, including *Arabidopsis thaliana* (72) [16], *Oryza sativa* (103) [17], *Zea mays* (120) [18], and *Solanum lycopersicum* (81) [19]. WRKY TFs share a conserved DNA-binding domain that contains a highly conserved WRKYGQK heptapeptide followed by a C2H2- or C2HC-type zinc finger motif [15, 20]. WRKY TFs function by recognizing and binding W-box *cis*-elements (TTGACC/T) of target genes, and both the heptapetide sequence and zinc finger motif are required for this high binding activity [15, 21, 22]. Based on the number of WRKY domains and the type of zinc fingers, WRKY TFs can be classified into three phylogenetically distinct groups: Group I WRKYs, which have two WRKY domains; Group II WRKYs, which have one WRKY domain, while both group I and II WRKYs contain one C2H2-type zinc finger motif (C-X4–5-C-X22–23-H-X1-H); and Group III members, which feature one WRKY domain and a C2HC-type motif (C-X7-C-X23-H-X1-C). Moreover, Group II is further divided into five subgroups (IIa-IIe) based on phylogenetic analyses [23–25].

WRKY TFs have been reported to be involved in many aspects of plant development [25, 26], including senescence [27, 28], trichome development [29], biosynthesis of secondary metabolites [21, 30–32], flowering [33, 34], and seed development and germination [35–37]. Substantial evidence has demonstrated that many *WRKY* genes also participate in various stress responses. For example, the expression of 18 *WRKY* genes was shown to be induced by exposure to salt stress in the roots of Arabidopsis [38]. *WRKY6* and *WRKY42* were identified to participate in the response to low Pi stress by regulating *PHO1* expression [39]. WRKY TFs from Arabidopsis were also shown to regulate the defence response positively and/or negatively against bacterial pathogens [16, 40], fungal pathogens [41–43] and nematodes [44, 45]. The expression levels of 13 *OsWRKY* genes from rice were examined in response to different treatments, including salt, polyethylene glycol (PEG), and cold or heat stresses, and 10 *WRKY* genes were down- or upregulated in response to these abiotic stresses. Moreover, WRKY proteins from tomato (*S. lycopersicum*) [19, 46], *Brassica napus* [47], soybean (*Glycine max*) [48], rice (*O. sativa*) [49, 50], wheat (*Triticum aestivum* L.) [51] and other plant species were shown to play critical roles in the response to various biotic and abiotic stresses.

According to the above mentioned discussion, WRKY TFs may participate in multiple pathways, leading to an array of physiological responses. The elucidation of the evolution and duplicative expansion of *WRKY* genes seems to be related to the diversity of their functions [20, 24]. The evolutionary studies of the *WRKY* gene family and large-scale genome-wide analyses of *WRKY* genes indicated that Group IIa genes, which compose the group with the fewest number of members, were the last to evolve and appear to have originated from Group IIb genes. Furthermore, Group IIa TFs play many important roles in the regulation of biotic and abiotic stress responses [20].

Cucumber (*Cucumis sativus* L.), one of the most economically important vegetable crop species, produces tender fruits that are edible organs [52, 53]. In addition, cucumber is extensively used as a model system in the study of sex determination, vascular biology, and induced defence responses [54]. In cucumber cultivation, yield and quality are frequently affected by different types of biotic and abiotic stresses, leading to a decline in cucumber output. Therefore, the identification of new functional genes for resistance to stresses is gaining considerable interest. Based on the cucumber genome (v1.0), 57 *WRKY* genes were identified, and 23 of them had been shown to be differentially expressed in response to at least one abiotic stress [55]. Low-coverage Sanger sequences and short high-coverage Illumina sequences were used to assemble draft cucumber genomes (v1.0 and v2.0); thus, these genomes are incomplete and of low quality. A high-quality and complete cucumber genome assembly (v3.0) is currently available for use in comparative genomics and genetic research [56]. Here, a new genome-wide identification of cucumber WRKYs was performed by the use of the cucumber (Chinese Long, 9930) genome (v3.0). We identified 61 *WRKY* genes and classified them into three groups. Comprehensive analyses including the gene structures, chromosomal locations, conserved protein domains, and phylogenetic analysis were further performed. The expression profiles of genome-wide *CsWRKY* genes in cucumber plants under different stresses were investigated. Our results will provide valuable clues for future work on the function of WRKYs in cucumber.

## Results

### The cucumber genome contains 61 *WRKY* genes

In a previous study, 57 *WRKY* genes were identified in the cucumber (Chinese long, 9930) genome (v1.0) [55]. Recently, an updated version (v3.0) was released in CuGenDB (http://cucurbitgenomics.org/), and the v1.0 was eliminated. Therefore, we identified cucumber *WRKY* genes in the cucumber genome (v3.0), and 61 *WRKY* genes were identified by a hidden Markov model (HMM) search using the WRKY domain (PF03106). These genes were proven to contain WRKY domains according to Pfam and SMART analysis. Among the previous 57 *WRKY* genes, five (*CsWRKY53*-*CsWRKY57*) were not conclusively mapped to any chromosome on the basis of the cucumber genome (v1.0) [55]; however, all 61 of the *WRKY* genes identified in this study could be mapped onto the chromosomes on the basis of the current version of the cucumber genome (v3.0) (Additional file 1: Fig. S1). There are seven chromosomes in the cucumber genome; the *WRKY* genes were not evenly dispersed across all chromosomes. Chromosome 3 harboured the highest number of *CsWRKY* genes (15, 24.59%), while only five (8.20%) were found on chromosome 5. Except for chromosomes 1 and 4, the number of *WRKY* genes we identified mapped onto every chromosome was at least one more than that in a previous study (Additional file 2: Fig. S2). Based on their order on the chromosomes, the *WRKY* genes identified in this study were renamed *CsWRKY1* to *CsWRKY61* (Additional file 1: Fig. S1), and this nomenclature approach was identical to that used in the previous study. A comparison of the currently known WRKY TFs in the cucumber genomes (Gy14, v1.0; 9930, v2.0 and v3.0) is listed in Additional file 3: Table S1.

For these 61 *WRKY* genes, the length of the coding DNA sequence (CDS) and the protein sequence, the protein molecular weight (MW), and the isoelectric point (pI) were analysed (Table 1 and Additional file 3: Table S1). The largest protein was CsWRKY8, comprising 1118 amino acids (aa), whereas the smallest one was CsWRKY47 (119 aa), corresponding to MWs ranging from 13.95 (CsWRKY47) to 124.59 (CsWRKY8) kDa. The pIs of the WKRYs ranged from 5.11 (CsWRKY10) to 10.08 (CsWRKY54). According to the predicted results of subcellular localization, all these CsWRKY proteins might be localized to the nucleus. The subcellular localization of CsWRKY50 (in this paper named CsWRKY47) [53] could support this claim.

**Table 1:** Features of *WRKY* genes identified in cucumber

Name	Gene ID (V3)	ORF	Intron number	AA	WRKY domain			Group
					Conserved heptapeptide	Zinc-finger type	Domain number	
**CsWRKY1**	**CsaV3_1G002180**	**1773**	**4**	**590**	**WRKYGQK**	**C2H2**	**1**	**II b**
**CsWRKY2**	**CsaV3_1G004720**	**1731**	**4**	**576**	**WRKYGQK/WRKYGQK**	**C2H2**	**2**	**I**
**CsWRKY3**	**CsaV3_1G007870**	**1845**	**3**	**614**	**WRKYGQK**	**C2H2**	**1**	**II b**
**CsWRKY4**	**CsaV3_1G028960**	**1521**	**4**	**506**	**WRKYGQK/WRKYGQK**	**C2H2**	**2**	**I**
**CsWRKY5**	**CsaV3_1G032000**	**849**	**2**	**282**	**WRKYGQK**	**C2H2**	**1**	**II d**
**CsWRKY6**	**CsaV3_1G033110**	**858**	**2**	**285**	**WRKYGQK**	**C2H2**	**1**	**II e**
**CsWRKY7**	**CsaV3_1G037680**	**1242**	**2**	**413**	**WRKYGQK**	**C2H2**	**1**	**II e**
**CsWRKY8**	**CsaV3_1G044520**	**3357**	**6**	**1,118**	**WRKYGQK/WRKYGQK**	**C2H2**	**2**	**I**
**CsWRKY9**	**CsaV3_2G013650**	**1047**	**2**	**348**	**WRKYGQK**	**C2H2**	**1**	**II d**
**CsWRKY10**	**CsaV3_2G017720**	**618**	**2**	**205**	**WRKYGKK**	**C2H2**	**1**	**II c**
**CsWRKY11**	**CsaV3_2G017760**	**741**	**3**	**246**	**WRKYGQK**	**C2H2**	**1**	**II c**
**CsWRKY12**	**CsaV3_2G032460**	**939**	**3**	**312**	**WRKYGQK**	**C2H2**	**1**	**II a**
**CsWRKY13**	**CsaV3_2G032470**	**612**	**3**	**203**	**WRKYGQK**	**C2H2**	**1**	**II a**
**CsWRKY14**	**CsaV3_2G034030**	**681**	**2**	**226**	**WRKYGQK**	**C2**	**1**	**II c**
**CsWRKY15**	**CsaV3_2G035630**	**882**	**2**	**293**	**WRKYGQK**	**C2H2**	**1**	**II d**
**CsWRKY16**	**CsaV3_3G003840**	**2244**	**4**	**747**	**WRKYGQK/WRKYGQK**	**C2H2**	**2**	**I**
**CsWRKY17**	**CsaV3_3G004410**	**1602**	**5**	**533**	**WRKYGQK/WRKYGQK**	**C2H2**	**2**	**I**
**CsWRKY18**	**CsaV3_3G007160**	**660**	**3**	**219**	**WRKYGQK**	**C2H2**	**1**	**II c**
**CsWRKY19**	**CsaV3_3G008170**	**1014**	**2**	**337**	**WRKYGQK**	**C2HC**	**1**	**III**
**CsWRKY20**	**CsaV3_3G008580**	**546**	**1**	**181**	**WRKYGQK**	**C2H2**	**1**	**II c**
**CsWRKY21**	**CsaV3_3G008610**	**1011**	**2**	**336**	**WRKYGQK**	**C2H2**	**1**	**II e**
**CsWRKY22**	**CsaV3_3G015290**	**972**	**2**	**323**	**WRKYGQK**	**C2H2**	**1**	**II c**
**CsWRKY23**	**CsaV3_3G018790**	**1521**	**4**	**506**	**WRKYGQK**	**C2H2**	**1**	**II b**
**CsWRKY24**	**CsaV3_3G021980**	**1101**	**2**	**366**	**WRKYGQK**	**C2HC**	**1**	**III**
**CsWRKY25**	**CsaV3_3G026600**	**999**	**3**	**332**	**WRKYGQK**	**C2H2**	**1**	**II c**
**CsWRKY26**	**CsaV3_3G026920**	**513**	**1**	**170**	**WRKYGQK**	**C2H2**	**1**	**II c**
**CsWRKY27**	**CsaV3_3G033000**	**990**	**1**	**329**	**WRKYGQK**	**C2HC**	**1**	**III**
**CsWRKY28**	**CsaV3_3G033350**	**1431**	**4**	**476**	**WRKYGQK/WRKYGQK**	**C2H2**	**2**	**I**
**CsWRKY29**	**CsaV3_3G035430**	**1419**	**4**	**472**	**WRKYGQK/WRKYGQK**	**C2H2**	**2**	**I**
**CsWRKY30**	**CsaV3_3G047140**	**1053**	**2**	**350**	**WRKYGQK**	**C2H2**	**1**	**II d**
**CsWRKY31**	**CsaV3_4G001260**	**873**	**2**	**290**	**WRKYGQK**	**C2H2**	**1**	**II e**
**CsWRKY32**	**CsaV3_4G003030**	**648**	**1**	**215**	**WRKYGQK**	**C2H2**	**1**	**II c**
**CsWRKY33**	**CsaV3_4G006110**	**810**	**1**	**269**	**WRKYGQK**	**C2H2**	**1**	**II e**
**CsWRKY34**	**CsaV3_4G006120**	**603**	**1**	**200**	**WRKYGQK**	**C2H2**	**1**	**II c**
**CsWRKY35**	**CsaV3_4G006480**	**1068**	**2**	**355**	**WRKYGQK**	**C2HC**	**1**	**III**
**CsWRKY36**	**CsaV3_4G025110**	**840**	**2**	**279**	**WRKYGQK**	**C2H2**	**1**	**II c**
**CsWRKY37**	**CsaV3_4G034570**	**1176**	**2**	**391**	**WRKYGQK**	**C2H2**	**1**	**II d**
**CsWRKY38**	**CsaV3_4G036610**	**981**	**4**	**326**	**WRKYGQK**	**C2H2**	**1**	**II a**
**CsWRKY39**	**CsaV3_5G001010**	**1035**	**2**	**344**	**WRKYGQK**	**C2H2**	**1**	**II e**
**CsWRKY40**	**CsaV3_5G011060**	**639**	**1**	**212**	**WRKYGQK**	**-**	**1**	**III**
**CsWRKY41**	**CsaV3_5G011080**	**849**	**2**	**282**	**WRKYGQK**	**C2HC**	**1**	**III**
**CsWRKY42**	**CsaV3_5G033090**	**1173**	**3**	**390**	**WRKYGQK**	**C2H2**	**1**	**I**
**CsWRKY43**	**CsaV3_5G038330**	**1521**	**3**	**506**	**WRKYGQK/WRKYGQK**	**C2H2**	**2**	**I**
**CsWRKY44**	**CsaV3_6G013820**	**921**	**2**	**306**	**WRKYGQK**	**C2H2**	**1**	**II c**
**CsWRKY45**	**CsaV3_6G018960**	**1287**	**4**	**428**	**WRKYGQK/WRKYGQK**	**C2H2**	**2**	**I**
**CsWRKY46**	**CsaV3_6G028510**	**1359**	**4**	**452**	**WRKYGQK**	**C2H2**	**1**	**II c**
**CsWRKY47**	**CsaV3_6G032100**	**360**	**3**	**119**	**WRKYGKK**	**C2**	**1**	**II c**
**CsWRKY48**	**CsaV3_6G032480**	**921**	**2**	**306**	**WRKYGQK**	**C2H2**	**1**	**II c**
**CsWRKY49**	**CsaV3_6G042200**	**735**	**2**	**244**	**WRKYGQK**	**C2H2**	**1**	**II c**
**CsWRKY50**	**CsaV3_6G042280**	**600**	**2**	**199**	**WRKYGKK**	**C2H2**	**1**	**II c**
**CsWRKY51**	**CsaV3_6G043450**	**885**	**2**	**294**	**WRKYGQK**	**C2H2**	**1**	**II d**
**CsWRKY52**	**CsaV3_6G048830**	**1872**	**5**	**623**	**WRKYGQK**	**C2H2**	**1**	**II b**
**CsWRKY53**	**CsaV3_6G051490**	**786**	**2**	**261**	**WRKYGQK**	**C2H2**	**1**	**II c**
**CsWRKY54**	**CsaV3_6G052610**	**1566**	**2**	**521**	**WRKYGQK**	**C2H2**	**1**	**II e**
**CsWRKY55**	**CsaV3_7G002670**	**1302**	**4**	**433**	**WRKYGQK/WRKYGQK**	**C2H2**	**2**	**I**
**CsWRKY56**	**CsaV3_7G003470**	**1608**	**4**	**535**	**WRKYGQK**	**C2H2**	**1**	**II-b**
**CsWRKY57**	**CsaV3_7G005750**	**897**	**2**	**298**	**WRKYGQK**	**C2H2**	**1**	**II e**
**CsWRKY58**	**CsaV3_7G022650**	**894**	**2**	**297**	**WRKYGQK**	**C2H2**	**1**	**II c**
**CsWRKY59**	**CsaV3_7G025370**	**891**	**2**	**296**	**WRKYGQK**	**C2HC**	**1**	**III**
**CsWRKY60**	**CsaV3_7G027490**	**768**	**3**	**255**	**WRKYGQK**	**C2H2**	**1**	**II d**
**CsWRKY61**	**CsaV3_7G030110**	**729**	**2**	**242**	**WRKYGQK**	**C2H2**	**1**	**II c**

### Multiple sequence alignment, phylogenetic relationship, and classification of CsWRKY proteins

**Fig. 1:**
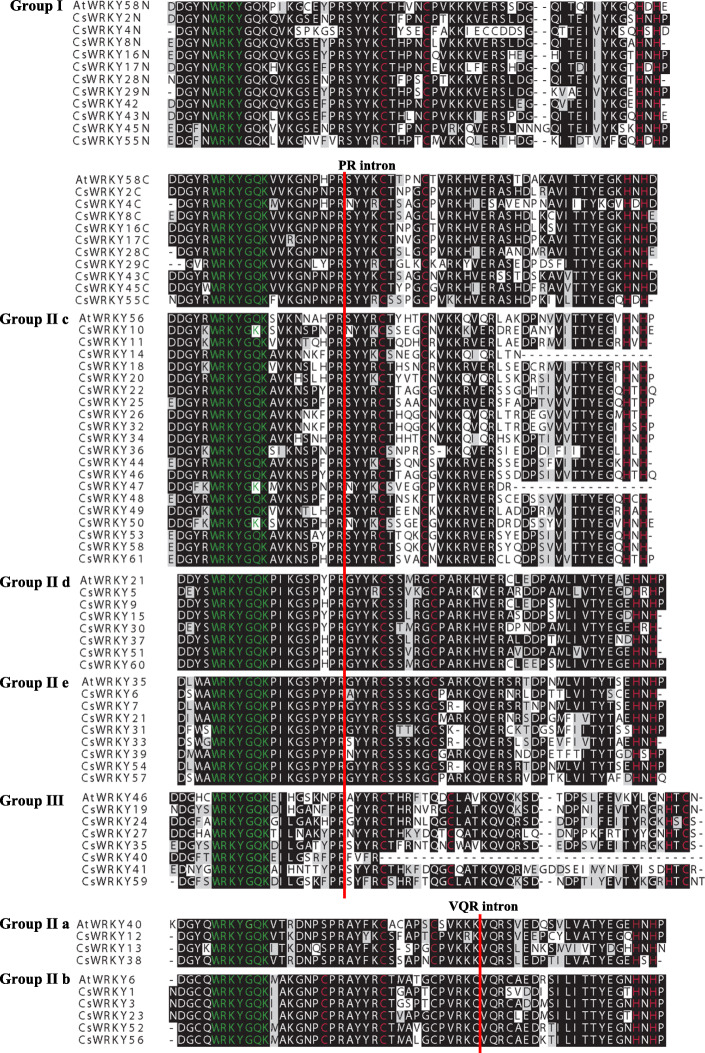
Alignment of 61 cucumber (CsWRKY) and 8 Arabidopsis (AtWRKY) WRKY domain sequences. For Group I WRKY proteins, N-terminal and C-terminal WRKY domains are represented by ‘N’ and ‘C’, respectively. The typical amino acid residues within WRKY domain and zinc-finger motif are in green and red color, respectively. The position of the intron in the genome is indicated by a red line for each WRKY subfamily

The WRKY domains, which comprise approximately 60 aa, of the newly identified CsWRKYs were first aligned, and seven AtWRKY domains (AtWRKY58, 56, 21, 35, 46, 40, and 6) from each group or subgroup were randomly selected as representatives for analysis. The highly conserved sequence WRKYGQK was found within a total of 58 CsWRKY proteins, while the others (CsWRKY10, CsWRKY47, and CsWRKY50) had a single amino acid substitution: K for Q (Fig. 1 and Table 1).

**Fig. 2:**
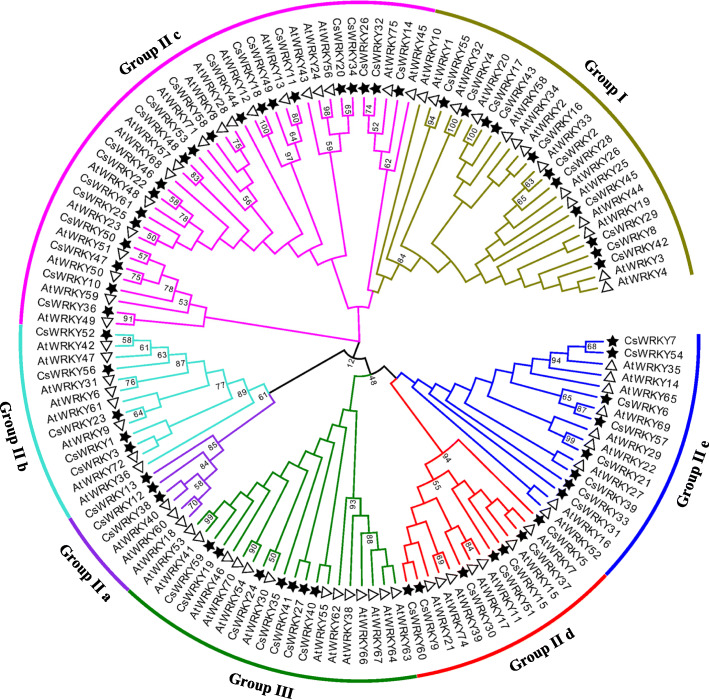
The phylogenetic tree of the total WRKY proteins from cucumber and Arabidopsis. The WRKY domains were used for phylogenetic analysis by MEGA5 software with bootstrap test of 1000 times. The arcs with different color represent 7 subgroups of WRKY proteins. The black solid star and hollow triangle represent WRKY domain from cucumber and Arabidopsis, respectively

A phylogenetic tree was constructed using the neighbour-joining (NJ) method by MEGA 5.0 software with 1000 bootstrap tests and based on multiple alignments of cucumber and Arabidopsis [16] WRKY domain aa sequences (Additional file 4: Table S2). As shown in Fig. 2, cucumber WRKY proteins could be categorized into three large groups (Group I-III) on the basis of the classifications of WRKYs in Arabidopsis [25]. Among the sequences of the 61 CsWRKY proteins, 11 sequences were assigned to Group I, 43 sequences belonged to Group II, and seven were assigned to Group III. In Group I, 10 members contained two WRKY domains (an N-terminal and a C-terminal WRKY domain), whereas CsWRKY42 had lost its C-terminal WRKYGQK-like stretch; these 11 members all harboured C2H2-type zinc finger motifs (C-X4-C-X22–24-H-X-H). The members of Group II contained a WRKY domain and could be further classified into five subgroups (IIa-IIe). Moreover, three members were classed in IIa, which was the group with the smallest number of members; 5, IIb; 20, IIc; 7, IId; and 8, IIe. Although most of the members of Group II had integral C2H2-type zinc finger motifs, partial absence of the zinc finger motif sequence was present in CsWRKY14 and CsWRKY47. Except for CsWRKY40, whose zinc finger motif was almost entirely absent, the CsWRKYs classed in Group III harboured a WRKY domain and contained a C2HC-type zinc finger motif. The ‘leucine-rich repeat’ (LRR) motif, which is a typical domain of resistance (R) proteins and is found in WRKY proteins of some species, such as Arabidopsis and rice, was not observed in the WRKY proteins of cucumber.

The Group IIa *WRKY* genes were found to be the last to evolve, as these genes compose the only group absent from the spike moss *Selaginella moellendorffii* [20]. The *WRKY* gene family members in many species have been identified, and their detailed numbers were listed in Table 2. Thus, we investigated the duplication and diversification of Group IIa *WRKYs* during evolution based on the available *WRKY* IIa genes in different species, including eight dicots (Arabidopsis, castor bean, cucumber, grape, tomato, pear, potato, and poplar) and six monocots (barley, rice, maize, bread wheat, *Brachypodium* and millet). The WRKY domain sequence of these *WRKY* IIa genes was used to construct a phylogenetic tree via MEGA 5.0 (Additional file 5: Table S3).

**Table 2:** Summary of the number of WRKY proteins in diverse plant species

Species	Name	Total	Group							NG	References
			I	IIa	IIb	IIc	IId	IIe	III		
*Ananas comosus*	AcWRKY	54	12	2	7	13	7	5	8	0	[22]
*Arabidopsis thaliana*	AtWRKY	72	13	4	7	18	7	9	14	0	[16]
*Brachypodium distachyon*	BdWRKY	86	15	3	6	21	6	10	23	2	[57]
*Brassica napus*	BnWRKY	300	78	11	34	55	28	30	51	13	[47]
*Cucumis sative*	CsWRKY	61	14	3	5	17	7	8	7	0	This study
*Glycine max*	GmWRKY	188	32	14	33	42	21	20	26	0	[48]
*Gossypium raimondii*	GrWRKY	112	20	7	16	26	16	13	14	0	[58]
*Gossypium arboreum*	GaWRKY	109	19	7	16	26	14	13	14	0	[58]
*Hordeum vulgare*	HvWRKY	45	8	4	1	11	5	3	13	0	[59]
*Manihot esculenta*	MeWRKY	85	17	5	14	20	8	9	12	0	[60]
*Musa acuminate*	MusaWRKY	153	14	7	11	15	15	13	6	72	[61]
*Oryza sativa*	OsWRKY	103a	15	4	8	15	7	11	36	0	[17]
*Populus trichocarpa*	PtrWRKY	100	22	5	9	27	13	13	10	1	[62]
*Pyrus bretschneideri*	PbrWRKY	103	17	6	10	24	15	16	15	0	[63]
*Ricinus communis*	RcWRKY	47	9	3	10	12	3	5	5	0	[64]
*Sesamum indicum*	SiWRKY	71	12	4	11	18	7	8	7	4	[65]
*Solanum lycopersicum*	SlWRKY	81	15	5	8	16	6	17	11	3	[19]
*Solanum tuberosum*	StWRKY	82	14	6	5	16	15	12	14	0	[66]
*Triticum aestivum*	TaWRKY	160	20	16	3	34	17	11	57	2	[51]
*Vitis vinifera*	VvWRKY	72	15	5	7	22	8	8	7	0	[67]
*Zea mays*	ZmWRKY	120b	15	6	8	22	13	16	30	0	[18]

a98 WRKY genes in japonica and 102 in indica rice. bother 10 *ZmWRKY* genes were classied in Group II. *NG no group*

**Fig. 3:**
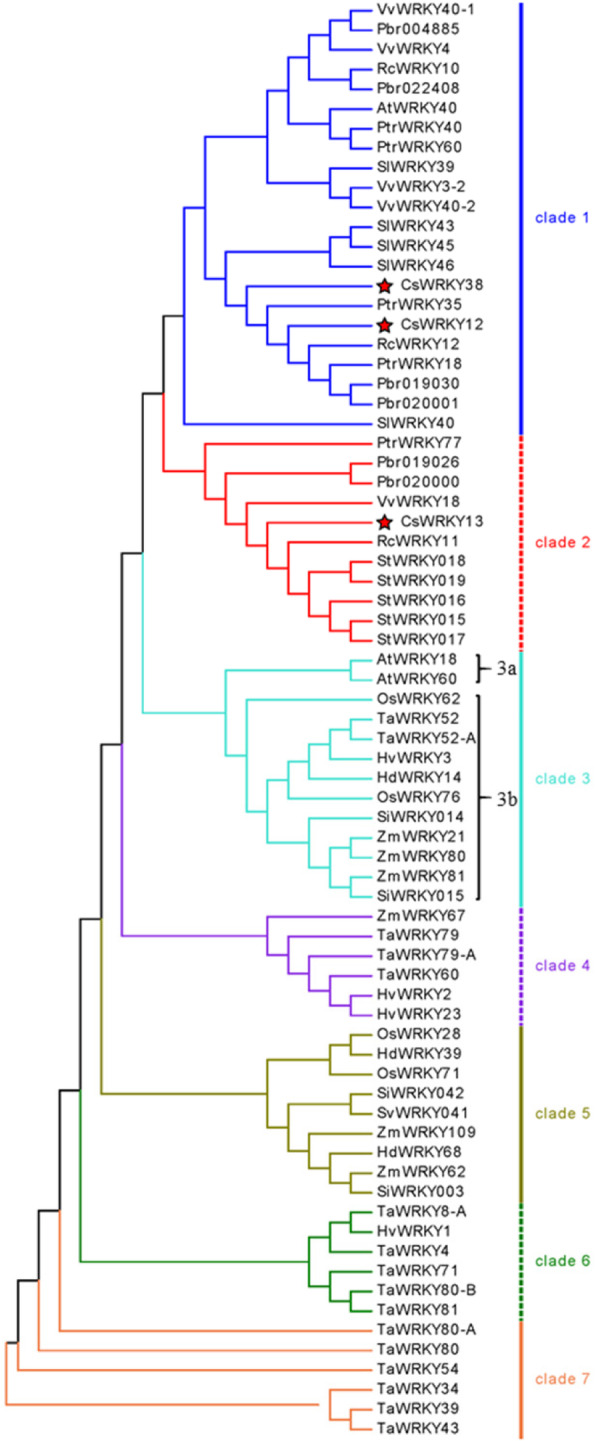
Phylogenetic clustering of group IIa WRKY proteins among fourteen different plant species. The phylogenetic tree was constructed by MEGA 5.0 using the Neighbor-Joining method. The WRKYs are classified into seven main clades with two subclades. The different-colored branch represents different clades. The red solid star indicates group IIa WRKY proteins from cucumber

As shown in the phylogenetic tree we constructed, the WRKY IIa proteins were categorized into seven clades (Fig. 3). WRKYs from the phylogenetically closer species clustered together in the same clade. For example, the members of clades 1 and 2 were all from dicots, whereas clades 4–7 contained proteins only from monocots; clade 3 was further divided into two different subclades (clades 3a and 3b) based on members from dicots or monocots, implying that the different evolutionary patterns of Group IIa WRKYs in dicots and monocots may have occurred after their divergence. WRKY members from one species clustered together at most within the three clades, and all the WRKY proteins that divided into three clades were from monocots. For the *WRKYs* from dicots, each of the four species (cucumber, grape, pear, and poplar) contributed at least one gene to clade 1 and clade 2; however, the three species (castor bean, tomato, and potato) clustered specifically within clade 1 or clade 2. These results suggested that numerous evolutionary splits and diversifications of *WRKYs* have occurred among different species.

### Gene structure and motif composition of *CsWRKYs*

**Fig. 4:**
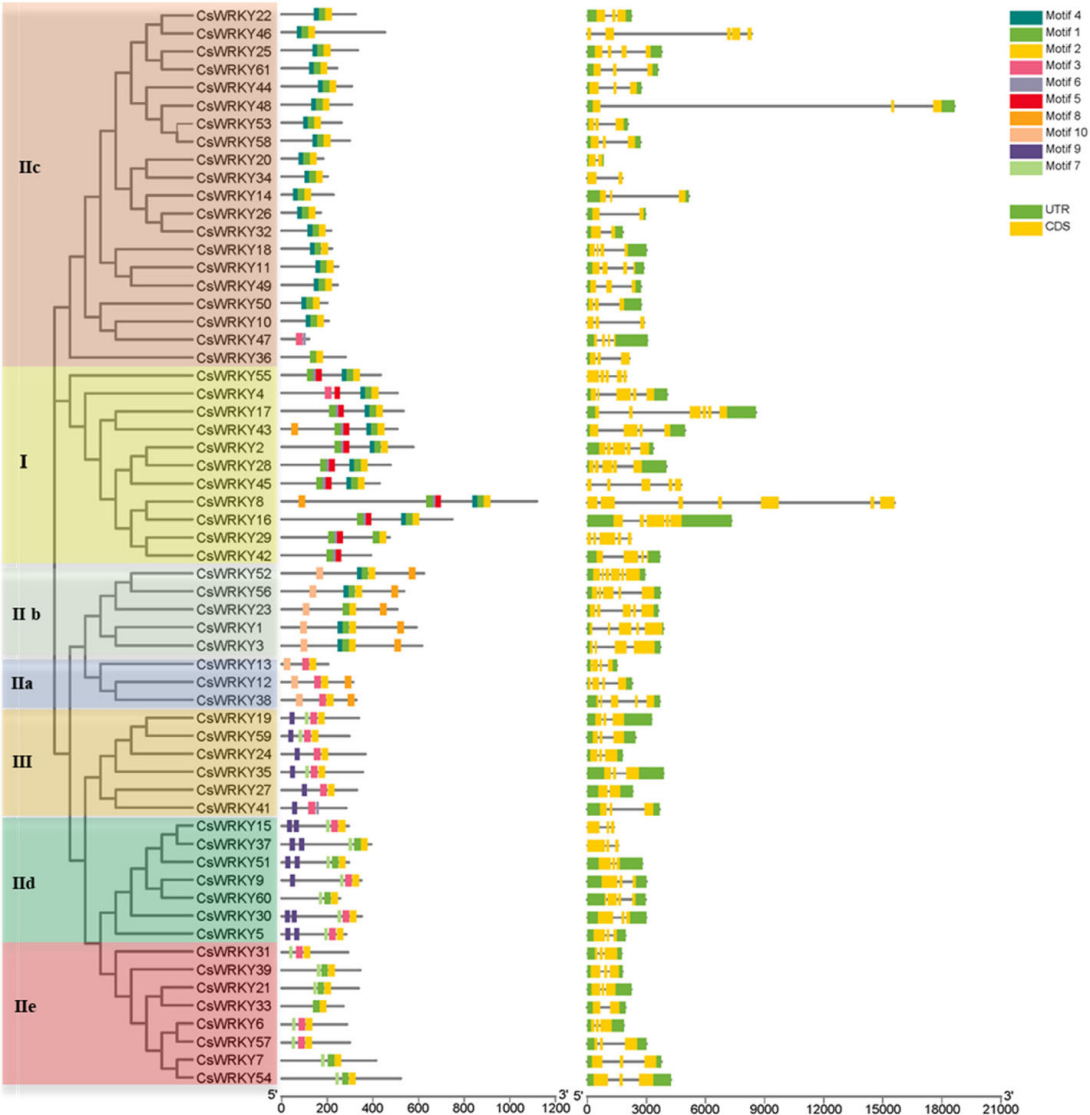
Phylogenetic clustering, conserved protein motifs and gene structure of *CsWRKY* genes. Left panel: the phylogenetic tree was constructed from the WRKY domain sequences of CsWRKY proteins. The different groups and subgroups are shown in different colors. Middle panel: the motifs are represented by different colored boxes. Details of each motif are shown in Additional file 5. Right panel: gene structure of *CsWRKY* transcription factors. Untranslated 5′- and 3′-regions, exons, and introns are indicated by green boxes, yellow boxes, and black lines, respectively

Gene structural diversity can reflect the evolution of multigene families [68]. Therefore, we analysed the exon-intron organization within the ORF (open reading fame) sequences of each *CsWRKY* gene (*CsWRKY40*, which lacked a zinc finger motif, was removed) to acquire more insight into the evolution of the WRKY family in cucumber. Previous studies showed that the majority of Populus and soybean WRKY members harboured two to four introns [48, 62]. Consistently, more than 80% of the members of the *CsWRKY* genes contain two to four introns (seven with one intron, 29 with two introns, 10 with three introns, 12 with four introns, two with five introns, and one with six introns) (Fig. 4 and Table 1). As shown in Fig. 4, a greater number of introns were observed in Group I, which varied from three to six. All WRKY domains typically contain an intron, and the position of this intron is extremely highly conserved [57]. We found that all *CsWRKYs* contained an intron in their WRKY domains. This intron within the Groups I (the C-terminal WRKY domain), IIc, IId, IIe, and III *WRKY* genes had the same location, which was after the codons for the invariant PR amino acid sequence (PR intron) (Fig. 1). The VQR intron, which occurs before the invariant VQR amino acid sequence, was observed in the Group IIa and IIb genes.

To better understand the conservation and diversification of *CsWRKYs*, the putative motifs of all CsWRKY proteins were predicted by MEME motif analysis. As expected, the CsWRKYs that were categorized into the same group shared highly similar motif compositions (Fig. 4 and Additional file 6: Table S4). For instance, motif 9 was found to be specific to Groups IId and IIe, whereas motif 10 was unique to Groups IIb and IIc; Groups IIe and IIc contained only two or three motifs, while Group IIb harboured 5 motifs. The functions of most of these motifs remain to be elucidated.

Overall, the closely related CsWRKYs in the phylogenetic tree shared similar gene structural and common motif compositions, suggesting that the CsWRKYs within the same group may play similar functional roles.

### Synteny analysis of *CsWRKY* genes

**Fig. 5:**
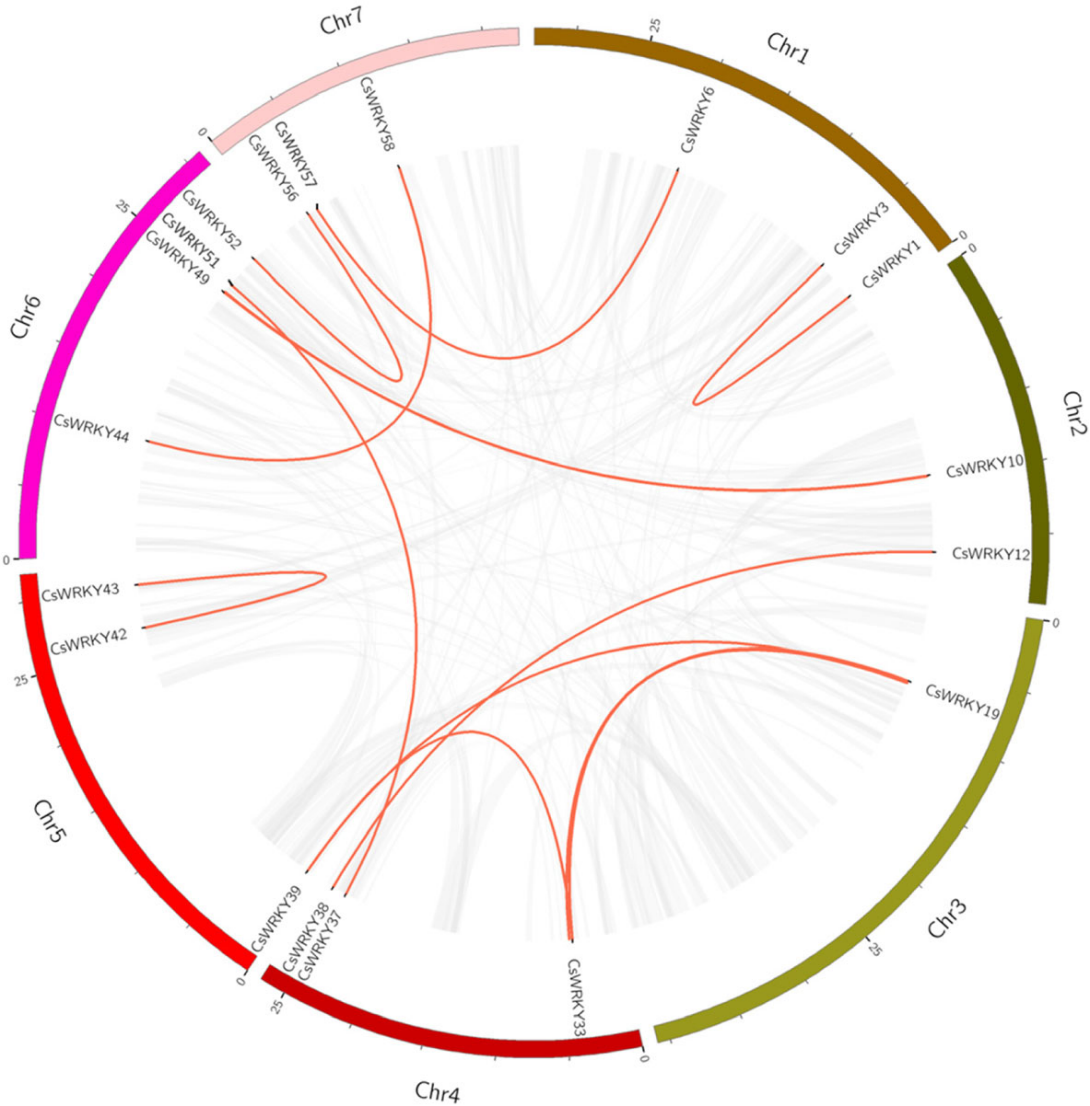
Schematic representations for the interchromosomal relationships of *CsWRKYs*. Gray lines show synteny blocks in the cucumber genome, duplicated *WRKY* gene pairs are connected with red lines

The segmental duplication events occurring in the cucumber WRKY family were investigated by conducting a synteny analysis of the *CsWRKY* genes using BLASTP and MCScanX. As shown in Fig. 5, 14 segmental duplication events involving 25 *WRKY* genes were observed (Additional file 7: Table S5). In contrast, tandem duplication events, which were defined by a chromosomal region within 200 kb containing two or more genes, were not identified for cucumber *WRKY* genes. These results suggested that some *CsWRKYs* were possibly generated by segmental duplication events and that the evolution of *CsWRKY* genes may have been driven, at least in part, by segmental duplication events.

**Fig. 6:**
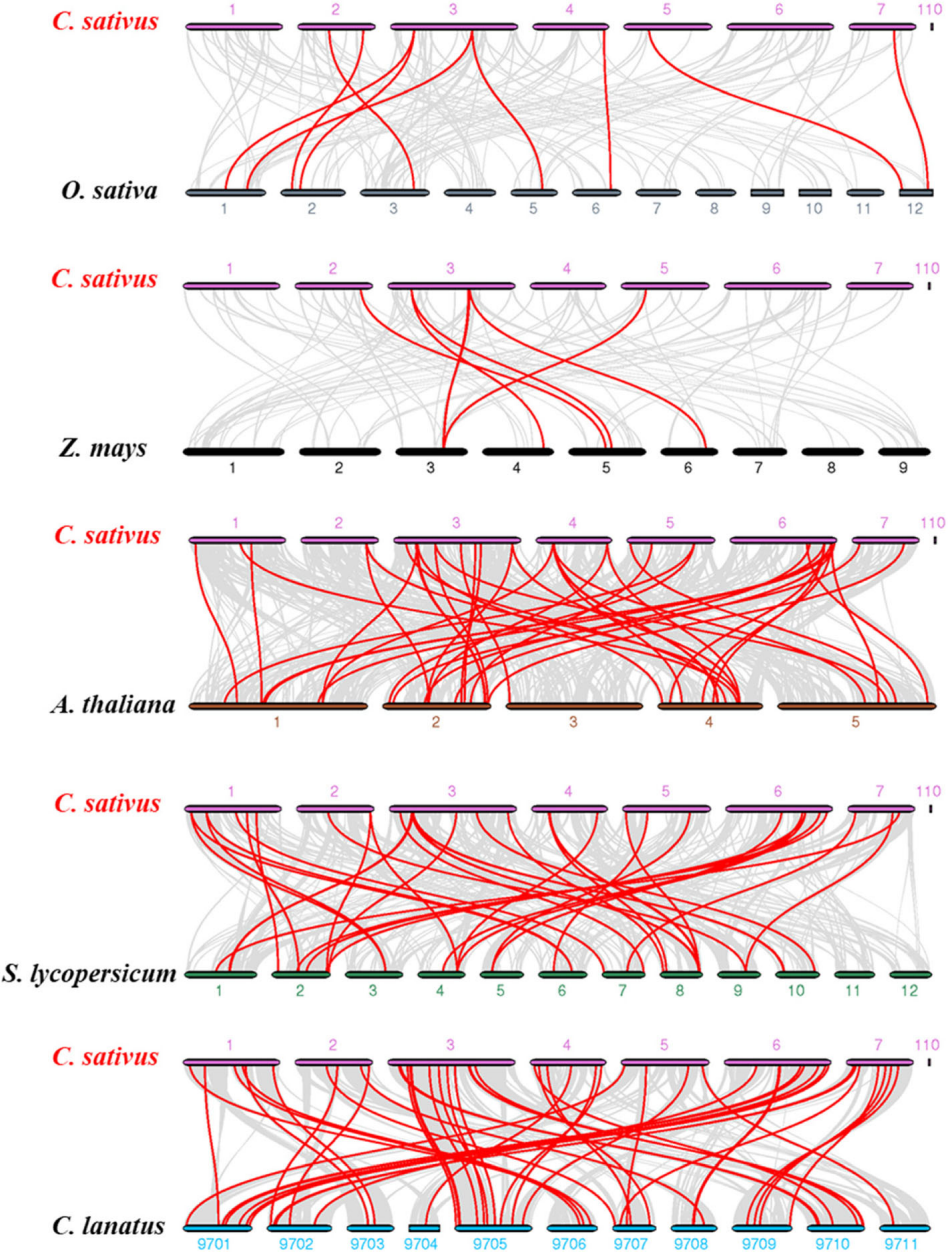
Synteny analysis of *WRKYs* between cucumber and other plant species. The collinear blocks are marked by gray lines, while the collinear gene pairs with *WRKY* genes are highlighted in the red lines. ‘*C. sativus*’, ‘*O. sativa*’, ‘*Z. mays*’, ‘*A. thaliana*’, ‘*S. lycopersicum*’ and ‘*C. lanatus*’ indicate *Cucumis sativus*, *Oryza sativa*, *Zea mays*, *Arabidopsis thaliana*, *Solanum lycopersicum*, and *Citrullus lanatus*, respectively

The phylogenetic mechanisms of the cucumber WRKY family were further explored by constructing comparative syntenic maps of cucumber associated with five representative species, including three dicots (Arabidopsis, tomato and watermelon) and two monocots (rice and maize) (Fig. 6). Fifty-two, 29, 27, 9, and 5 *CsWRKY* genes showed syntenic relationships with those in the other five species: watermelon, tomato, Arabidopsis, rice and maize, respectively. A total of 52 *WRKY* collinear gene pairs between cucumber and watermelon were identified, followed by cucumber and Arabidopsis (41), cucumber and tomato (37), cucumber and rice (9), and cucumber and maize (7) (Additional file 8: Table S6). Both cucumber and watermelon belong to the gourd family, and more than 85% of the *CsWRKY* genes showed a syntenic relationship with *WRKY*s in watermelon, and one *CsWRKY* gene was associated with only one syntenic gene pair, indicating that *WRKY* genes in cucumber and watermelon evolved from the same ancient *WRKY* genes. *CsWRKY21* and *CsWRKY28* were found to be associated with two syntenic gene pairs between cucumber and tomato/rice/maize; some *CsWRKY* genes were associated with three collinear gene pairs (between cucumber and tomato/Arabidopsis *WRKY* genes), speculating that these *CsWRKYs* may play an important role in the evolution of the *WRKY* gene family. Importantly, collinear *CsWRKY21* gene pairs were observed between cucumber and all of the other five species, suggesting that this orthologous pair may have formed before the divergence of dicotyledonous and monocotyledonous plants.

### *CsWRKYs* expression profiles in different organs

**Fig. 7:**
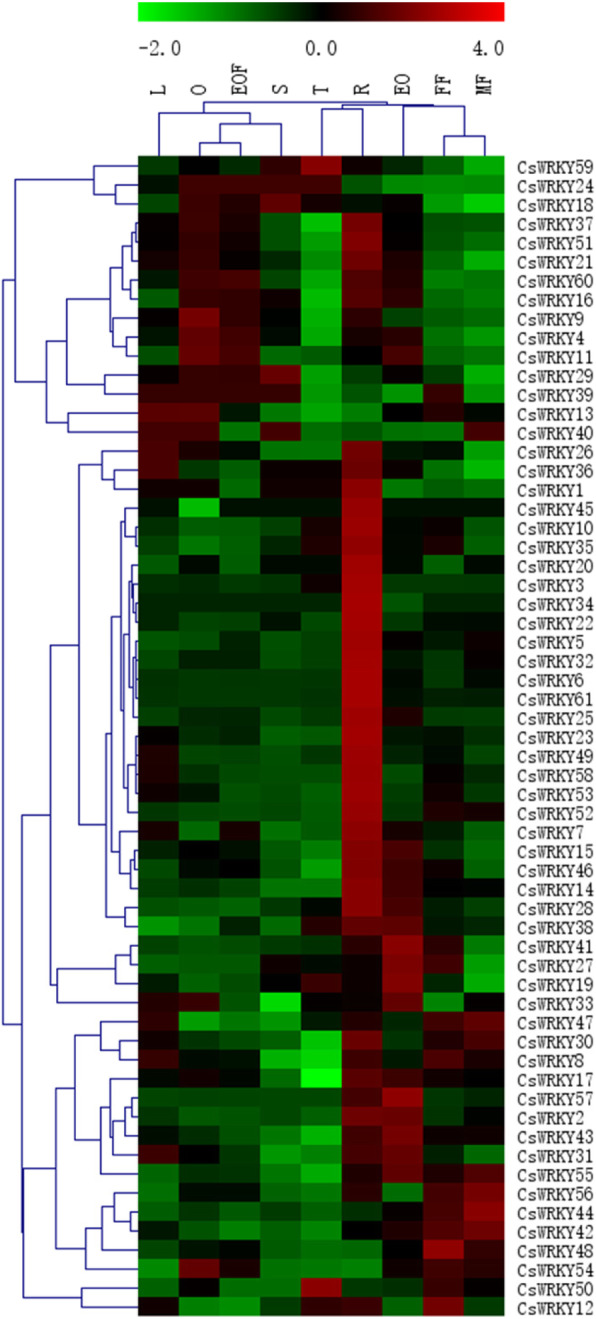
Tissue-specific expression of *WRKY* genes in cucumber. The transcriptional levels of *CsWRKY* genes in nine tissues of cucumber 9930 were investigated based on a public transcriptome data, and only one biological replication was used for each tissue sample [69]. The genome-wide expression of *CsWRKY* genes was shown on a heatmap using FPKM value, and the expression data were gene-wisely normalized by MeV (Multiple Experiment Viewer) software. -2.0 to 4.0 was artificially set with the color scale limits according to the normalized value. The color scale shows increasing expression levels from green to red. L, leaves; O, ovary; EOF, expanded fertilized ovary; S, stem; T, tendril; R, root; EO, expanded unfertilized ovary; FF, female flower; MF, male flower

The expression patterns of all 61 *CsWRKYs* were investigated using a standard transcriptome analysis procedure based on public transcriptomic data of different tissues of cucumber, including roots, stems, leaves, female flowers, male flowers, ovaries, expanded unfertilized ovaries, expanded fertilized ovaries, and tendrils [69]. Among the 61 *CsWRKY* genes, 41 *CsWRKYs* were expressed in all detected samples (FPKM> 0), and 24 genes showed constitutive expression (FPKM> 1 in all samples) (Additional file 9: Table S7). Some *CsWRKY* genes showed preferential expression across all tissues tested. Nineteen genes in the roots, two genes in the tendrils (*CsWRKY50*/*59*), and two genes in the female flowers (*CsWRKY48/12*) exhibited the highest transcript levels. The expression analysis of the different fruit developmental stages showed that several genes (*CsWRKY9/40/54*) had higher expression in the ovaries than in the expanded ovaries (fertilized and unfertilized). In addition, the transcript levels of some *CsWRKYs* (such as *CsWRKY19/27/41/57*) decreased in the fertilized expanded ovaries (Fig. 7). These results indicated that these genes may play roles in many aspects of cucumber plant development, including ovary development and fruit fertilization.

### Expression patterns of *CsWRKYs* in response to abiotic and biotic stresses

To confirm whether *CsWRKYs* are involved in the response to various stresses, we analysed the comprehensive expression patterns of *CsWRKY* genes under different abiotic and biotic stresses, including salt, heat, downy mildew (DM, *Pseudoperonospora cubensis*) and powdery mildew (PM, *Podosphaera fusca*), based on public transcriptome information [54, 70, 71] and transcriptomic data that we generated.

**Fig. 8:**
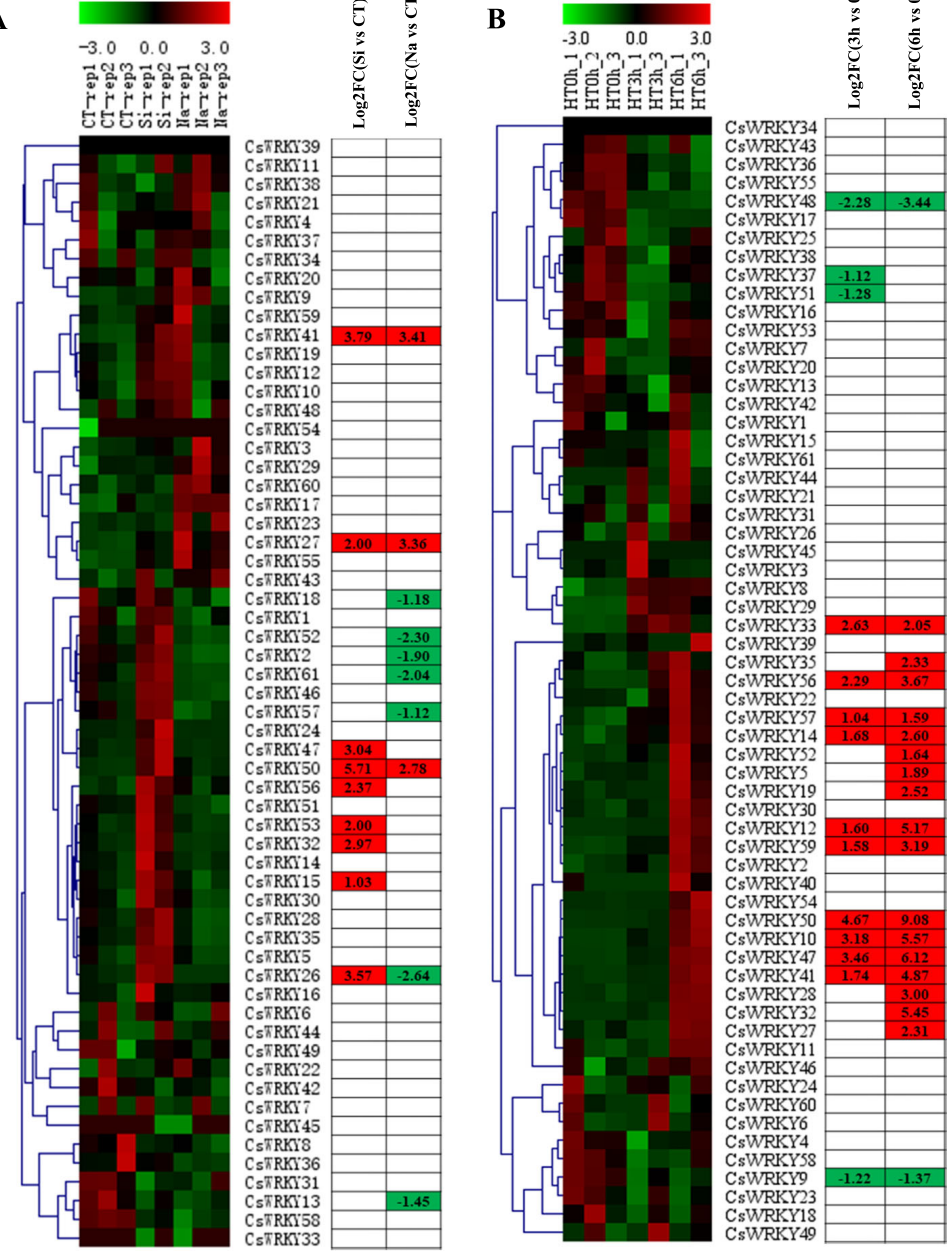
Expression profiles of *CsWRKY* genes in response to various abiotic stress treatments. The transcriptional levels of *CsWRKY* genes in response to salt (**a**) and heat (**b**) stresses were investigated based on a public transcriptome data [70] and transcriptome data that we performed, respectively. The genome-wide expression of *CsWRKY* genes under different treatments was shown on a heatmap using FPKM values, and the expression data were gene-wisely normalized by MeV software. -3.0 to 3.0 was artificially set with the color scale limits according to the normalized value. The color scale shows increasing expression levels from green to red. In the table, differentially expressed genes (DEGs) were highlighted by red (up-regulation) and green (down-regulation). FC, fold-change; CT, control; HT, heat treatment; HT0h = heat treatment for 0 h (hours); HT3h = heat treatment for 3 h; HT6h = heat treatment for 6 h

To investigate the potential functions of *CsWRKYs* in resistance to salt stress, we performed a *CsWRKYs* expression analysis after salt treatment based on public transcriptomic data [70] (Additional file 10: Table S8). We observed that the expression levels of *CsWRKY27, CsWRKY41* and *CsWRKY50* considerably increased in response to salt stress. Moreover, seven genes exhibited the opposite trend with exposure to salt stress (Fig. 8a). Previous studies have shown that Silicon (Si) application can improve plant growth under salt stress [72]. Among the seven genes that were downregulated under salt stress, the expression levels of six genes reverted to normal expression levels, and the DEGs (differentially expressed genes) were all upregulated in response to exogenous Si treatment, implying a potential role of these *WRKY* genes in the Si-based alleviation of salt stress (Fig. 8a). Furthermore, the expression patterns of all 61 *CsWRKY* genes in the transcriptomic data, which were derived from leaves subjected to different heat treatment durations, were investigated in this study (Additional file 10: Table S8). Correlation and cluster analyses were used to explore the similarity among the transcriptomes. Two samples (HT3h_2 and HT6h_2) were removed due to their poor uniformity, and the remaining seven samples were used for the following analysis (Additional file 11: Fig. S3). As shown in Fig. 8b, 21 *CsWRKY* genes were significantly induced/repressed by heat stress. The variation trend of the expression of most *WRKY* genes in response to heat stress for 3 h (hours) was consistent with that for 6 h. Overall, the transcript levels of five *CsWRKY* genes (*CsWRKY27/41/50/52/57*) were significantly affected by both salt and heat stress treatments.

**Fig. 9:**
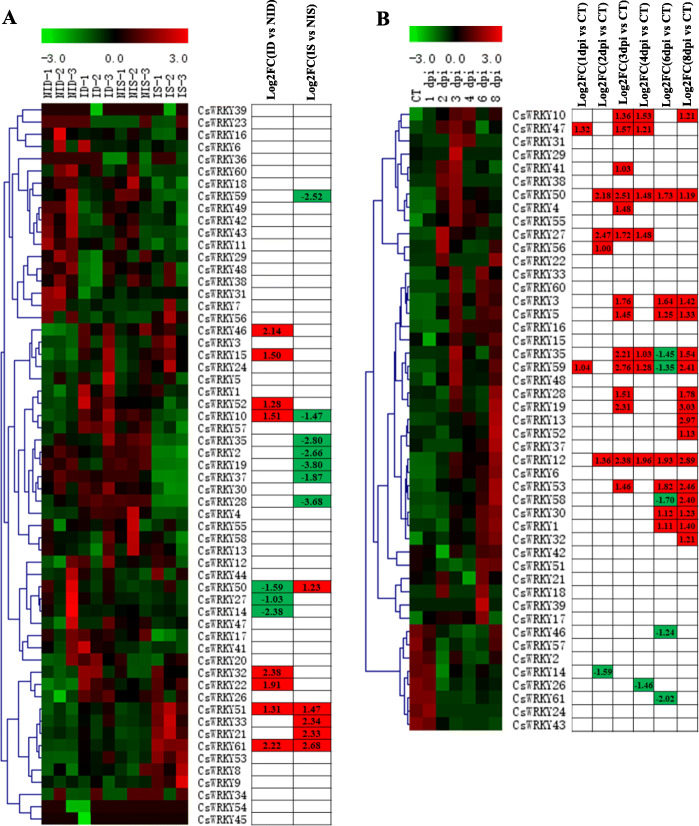
Expression analysis of *CsWRKYs* under biotic stresses. The *CsWRKY* genes transcripts were determined after the infection of powdery mildew (PM) for 48 h (**a**) [71] and of downy mildew (DM) for 1 to 8 days post inoculation (**b**) [54], respectively. Without inoculation as the control (CT). The FPKM values of *CsWRKY* genes under different treatments were gene-wisely normalized and shown on a heatmap by MeV software. -3.0 to 3.0 was artificially set with the color scale limits according to the normalized value. The color scale shows increasing expression levels from green to red. In the tables, red shadings indicated up-regulated genes, green shading indicated down-regulated genes. FC, fold-change; ID, DM inoculated susceptible cucumber line D8 leaves; NID, non-inoculated D8 leaves; IS, DM inoculated resistant cucumber line SSL508–28 leaves; NIS, non-inoculated SSL508–28 leaves; DPI, day post inoculation

To explore the potential functions of *CsWRKYs* in resistance to biotic stresses, we performed a *CsWRKYs* expression analysis of the susceptible and resistant cucumber lines inoculated with PM for 48 h based on published RNA-seq data [71] (Fig. 9a and Additional file 10: Table S8). Eleven and 12 *CsWRKY* genes that were differentially expressed were identified in the susceptible and resistant cucumber lines, respectively, compared with the controls. These results indicated that these *WRKYs* may be influenced by PM stress. The expression patterns of *CsWRKY10* and *CsWRKY50* were opposite in the susceptible and resistant cucumber lines under inoculation with PM, implicating the important role of these two *WRKY* genes in the response to PM infection. The expression of *CsWRKY* genes in response to DM infection was obtained by transcriptome analysis based on RNA-seq data published by Adhikari et al. [54] (Additional file 10: Table S8). Twenty-five *CsWRKY* genes in cucumber were involved in responses to DM infection, indicating that they were induced to play a role in response to DM infection (Fig. 9b). We identified 12 *CsWRKY* genes (*CsWRKY10/14/19/27/28/32/35/46/50/52/59/61*) that were differentially expressed in response to the inoculation of PM and DM (Fig. 9), indicating that these genes may play key roles in responses to biotic stresses. Some *CsWRKY* genes were affected only by inoculation of PM and/or DM and not by abiotic (heat and salt) stresses (Figs. 8 and 9). For instance, *CsWRKY46* was expressed significantly in response to inoculation of PM and DM but not to salt and heat stresses; moreover, *CsWRKY15* was not induced/repressed by any of the tested treatments except inoculation of PM. In addition, the expression levels of several *CsWRKY* genes were significantly affected by both abiotic stresses and biotic stresses (Figs. 8 and 9). For instance, *CsWRKY27*, *CsWRKY50* and *CsWRKY52* simultaneously responded to all treatments analysed, and the expression of *CsWRKY59* was affected by all tested treatments except salt treatment.

## Discussion

Although *WRKY* genes had been identified in the cucumber (9930) genome (v1.0) [55], it is essential to re-identify them. Because the information of the *WRKY* genes identified in v1.0 were no longer available, due to the elimination of v1.0 from CuGenDB (http://cucurbitgenomics.org/), whereas the assemblies (v2.0 and v3.0) were available. Therefore, we identified and characterized the WRKY family in the cucumber (9930) genome (v3.0). It is composed of 61 members, which were designated CsWRKY1 to CsWRKY61 on the basis of their chromosomal location; this number is higher than that identified in a previous study (57 *WRKY* genes) [55]. Compared with these previously reported *CsWRKY* genes, nine new *CsWRKY* genes were mapped onto the chromosomes, and five previous *CsWRKY* genes that could not be conclusively mapped to any chromosome were considered obsolete according to the current version of the cucumber genome (v3.0) (Additional file 1: Fig. S1 and Additional file 2: Fig. S2).

Based on the gene structures, amino acid sequences, conserved structural domains and phylogenetic relationships with *A. thaliana*, the 61 CsWRKY proteins were similar to the typical WRKY family proteins in other species with classifications into Groups I, IIa, IIb, IIc, IId, IIe and III (Table 1, Figs. 1 and 2). Rinerson et al. [20] proposed that there were four major WRKY TF lineages in flowering plants, Groups I + IIc, Groups IIa + IIb, Groups IId + IIe, and Group III, accurately reflecting the evolution of the WRKY family. This was also verified in cucumber; for example, the members of Groups IIa and IIb (or Groups IId and IIe) were divided into two subclades, which involved the same clade; some WRKY TFs from Group IIc were classified into one subclade together with the members of Group I (Fig. 2). Three CsWRKY proteins (CsWRKY10/47/50) in Group IIc showed sequence variation in their WRKY domains. Domain loss, which seems to be common in monocotyledons, is one of the divergent forces for expansion of the *WRKY* gene family [73, 74]; however, these loss-of-domain events occur less for dicotyledons than for monocotyledons. For example, Group I contains one protein (AtWRKY10) having only one WRKY domain in Arabidopsis [74]. In cucumber, except CsWRKY42, all WRKY proteins of Group I have two WRKY domains, and the event by which the zinc finger motif was lost was also found in three WRKY TFs (CsWRKY14/40/47) (Fig. 1 and Table 1). According to previous studies, both the heptapeptide motif WRKYGQK and the zinc finger motif are required for the high binding affinity of WRKY TFs to their cognate *cis*-acting W-box element (TTGACC/T). Therefore, variations in the heptapeptide motif and loss of the zinc finger motif might influence normal interactions of CsWRKYs with target genes, and it might be worth further studying the binding specificities and functions of these five CsWRKY proteins.

Both tandem duplication and chromosomal/segmental duplications contributed to the expansion of the *WRKY* gene family [24]. Comparison of the number of *WRKY* genes in the cucumber genome with other sequenced dicotyledon genomes showed that cucumber has fewer genes (Table 2). Fourteen segmental duplication events within 25 *WRKY* genes were observed (Fig. 5 and Additional file 7: Table S5), while tandem duplication events were lacking. Therefore, the lack of tandem duplication might be a possible reason for the smaller number of *CsWRKY* genes, and segmental duplication was a major driver of *WRKY* gene expansion during the cucumber evolutionary process. Moreover, we identified that more than 85% (52 of 61) of *CsWRKY* genes showed orthologous relationships with *ClWRKY* genes (Additional file 8: Table S6), indicating that the segmental duplication of *WRKY* genes might have occurred in diploid progenitors before the divergence of the cucumber and watermelon.

In 2015, Rinerson et al. used the genome sequences of a moss to propose the hypothesis that Group III genes were not the last to evolve; rather, Group IIa genes were [20]. Among the *WRKY* gene family, Group IIa genes compose the subclade with the smallest number of members and appear to play many important roles in the response to different stresses [20]. The availability of increased numbers of Group IIa members of sequenced plant genomes could provide additional clues about the evolution of the WRKY TF family. In this study, we found that members of the plant WRKY Group IIa from closely related species tended to be clustered together, and there were monocot (clades 1, 2 and 3a)- and dicot (clades 3b and 4–7)-specific clades (Fig. 3). These results suggested that *WRKY* IIa genes might have evolved independently after the divergence of monocots and dicots.

It is well known that gene expression is correlated with gene function [75]. In this study, the expression pattern of all 61 *CsWRKY* genes was analysed in nine different tissues of cucumber, including the roots, stems, leaves, flowers, ovaries, and tendrils. We found that 19 *CsWRKY* genes were expressed specifically in the roots (Fig. 7). As previously reported, *AtWRKY23*, *AtWRKY75* and *AtWRKY6* were found to regulate root development [76, 77], and their close genetic homologous genes in cucumber, *CsWRKY25*, *CsWRKY32* and *CsWRKY52*, respectively, were expressed specifically in the roots. According to these results, all genes expressed specifically in the roots were assumed to be key regulators of root development and may play roles in response to various stresses that first affect plants below ground. Additionally, *CsWRKY50* and *CsWRKY59* were highly expressed in the tendrils, which are considered abnormal leaves in cucumber, implying that they may regulate leaf morphogenesis in cucumber. Noticeably, the expression profiles of cucumber *WRKY* genes in this study were not consistent with the results in the study of Ling et al. [55]. For example, *WRKY18* and *WRKY56* have extremely different patterns of expression profiles between these two studies (Fig. 7) [55]. The reason might be that although they have the same name, they actually are different genes. This prediction could be supported by that *WRKY56* could not be conclusively mapped to any chromosome in the previous study [55], but to chromosome 7 in our study (Additional file 1: Fig. S1), and that *WRKY18* was localized in different position on chromosome 3 (Additional file 1: Fig. S1) [55]. However, now it is very difficult to clarify the reason because that the v1.0 of the cucumber genome sequences is not available yet, and Ling et al. did not provide the gene or protein sequences of WRKYs in their paper [55].

WRKY proteins constitute one of the most important TF families and are involved in responses to biotic and abiotic stresses [25]. At least 26 and 54 *WRKY* genes were identified to respond to abiotic stress in Arabidopsis and rice, respectively [38, 78]. In this study, we further explored the expression of 61 *CsWRKY* genes under multiple stresses. Most of them were induced/repressed by at least one of the stresses that we tested (heat, salinity, and inoculation of DM and PM), indicating that the *CsWRKYs* play crucial roles in cucumber stress responses. Four *CsWRKY* genes, *CsWRKY9*, *CsWRKY18*, *CsWRKY48* and *CsWRKY57*, were responsive to heat and/or salinity stress but not to the inoculation of DM and PM (Figs. 8 and 9). Previous studies have revealed that one *WRKY* gene can function in response to several stresses. For example, overexpressing *AtWRKY30* improved tolerance to oxidative and salinity stresses during seed germination [79]. Among these four genes, the transcript level of *CsWRKY57* was significantly affected by both salt and heat stress treatments (Fig. 8), suggesting that this gene acts as the most important gene to regulate susceptibility to abiotic stresses in cucumber. Correspondingly, 10 *CsWRKY* genes (*CsWRKY1/3/4/15/21/22/30/46/53/58*) were affected only by inoculation of PM and/or DM and not by abiotic (heat and salt) stresses (Figs. 8 and 9), and only *CsWRKY46* was observed to be differentially expressed in response to inoculation of PM and DM. These results revealed significant differences in the stress-induced expression of *WRKYs* in response to abiotic and biotic stresses. In addition, 22 *CsWRKY* genes were significantly affected by both abiotic stresses and biotic stresses (Figs. 8 and 9), indicating that some *CsWRKY* genes have similar functions in response to both abiotic and biotic stresses. For instance, the expression of *CsWRKY59* was affected by all tested treatments except salt treatment; *CsWRKY27*, *CsWRKY50* and *CsWRKY52* simultaneously responded to all treatments that we analysed. The expression of *OsWRKY67* was activated by rice blast inoculation; overexpression of *OsWRKY67* in rice plants enhanced resistance to leaf blast, panicle blast and bacterial blight [80]; and its orthologue in cucumber, *CsWRKY50*, was also induced by biotic stresses, suggesting the potential value of *CsWRKY27*, *CsWRKY50* and *CsWRKY52* in improvements to cucumber abiotic and biotic stress tolerance. Moreover, the expression of 12 *CsWRKY* genes (*CsWRKY6/16/17/24/29/31/38/39/42/43/55/60*) was not observed in response to either the biotic stresses or abiotic stresses we analysed in this study.

As shown in Fig. 9, the expression of *CsWRKY19* was downregulated by PM infection but upregulated by DM. The results indicated that *WRKY* genes might play different roles under different stress responses. Further analysis showed that responses to stresses occurred at different timepoints. *CsWRKY10* and *CsWRKY47* responded to heat stress at 3 h, whereas the expression of *CsWRKY28* and *CsWRKY35* was affected at 6 h after heat stress began; *CsWRKY56* was highly expressed only at 2 dpi, while infection by DM upregulated the expression of *CsWRKY12* and *CsWRKY50* between 2 to 8 dpi, suggesting that *CsWRKY* genes might play important regulatory roles at different stages in cucumber abiotic and biotic stress tolerance.

Overall, these above findings provide insights into the potential functions of cucumber *WRKY* genes. The differential expression in response to different stresses indicated their functional diversification. Some *CsWRKY* genes might specifically respond to biotic or abiotic stress, while several genes may respond to both biotic and abiotic stress. In addition, some *CsWRKY* genes might not be involved in stress responses. These results are helpful for future functional characterization of *CsWRKY* genes and for the genetic improvement of the abiotic and biotic stress resistance of cucumber.

## Conclusions

In the present study, 61 cucumber *WRKY* genes were identified, and a comprehensive analysis of those *CsWRKY* genes was carried out. First, the chromosomal location, conserved motifs, evolutional relationships and gene structure of the cucumber *WRKY* genes were examined. The expression patterns of the *CsWRKY* genes in nine different tissues of cucumber cultivar 9930 and in response to various stresses then showed that these genes may play important roles in cucumber growth and development. Furthermore, our results revealed differences and similarities in the stress-induced expression of *CsWRKYs* in response to abiotic and biotic stresses. In conclusion, our study provided a foundation for future studies into the functions of *WRKY* genes important in responses to abiotic and biotic stresses and the identification of new sources of resistance for breeding programmes.

## Methods

### Gene identification and chromosomal locations

The hidden Markov model (HMM) file of the WRKY domain (PF03106), downloaded from the Pfam protein family database (http://pfam.sanger.ac.uk/), was used for the identification of *WRKY* genes from the cucumber genomic database (v3.0) by HMMER 3.0. The default parameters were employed, and the cutoff value was 0.01. All *CsWRKY* genes that were queried from the cucumber genomic data based on the HMMER results were further examined to confirm the existence of the WRKY domain sequences through the Pfam (http://pfam.xfam.org/search#tabview=tab1) and SMART (http://smart.embl-heidelberg.de/) databases. We then manually examined each candidate gene to ensure the conserved heptapeptide sequence within the predicted WRKY domain and used PCR amplification and sequencing to further validate select *CsWRKY* genes. Sixty-one *WRKY* genes were ultimately identified and mapped to cucumber chromosomes according to their physical location information from the cucumber genomic database. The subcellular localization of CsWRKY proteins was predicted using CELLO (http://cello.life.nctu.edu.tw/).

### *CsWRKY* genes structure analysis, classification and phylogenetic analysis

The gene structures of all identified cucumber WRKY genes were identified by the Gene Structure Display Server (GSDS, http://gsds.cbi.pku.edu.cn/). The cucumber *WRKY* genes were classified into different groups according to the classification scheme of Arabidopsis *WRKY* genes and the WRKY domain alignments of CsWRKY and AtWRKY proteins. Alignments of the amino acid sequences of the following were performed using ClustalX with default settings: WRKY domains from cucumber and Arabidopsis (excluding the C-terminal domains of Group I); 61 full-length CsWRKYs; and the Group IIa WRKY domains from Arabidopsis [16], castor bean [64], cucumber, grape [67], tomato [19], pear [63], potato [66], poplar [62], barley [59], rice [17], maize [18], bread wheat [51], *Brachypodium* [57] and millet [81]. Phylogenetic trees were then constructed based on the alignments using the neighbour-joining (NJ) method of MEGA 5.0. The trees were visualized and optimized via Evolview (http://www.evolgenius.info/evolview).

### Motif composition analysis of CsWRKY proteins

The motifs within the 61 cucumber WRKY protein sequences were identified using the MEME online program (http://meme.nbcr.net/meme/intro.html) with the following parameters: number of repetitions, any; maximum number of motifs, 10; and optimum width of each motif, between 6 and 300 residues.

### Analysis of gene duplication

The Multiple Collinearity Scan toolkit (MCScanX) was used to examine the gene duplication events, with the default parameters [82]. To explore the syntenic relationships of the *WRKY* genes obtained from cucumber and other selected species, syntenic analysis maps were constructed using MCScanX.

### Regulatory elements in the promoter regions of *CsWRKY* genes

The elements in the 1.5 kb promoter fragments (upstream sequences of the CsWRKY-encoding sequences) of the *CsWRKY* genes were analysed using the online PlantCARE database (http://bioinformatics.psb.ugent.be/webtools/plantcare/html/).

### Transcriptome analysis of *WRKY* genes in cucumber

The expression patterns of the *CsWRKYs* were analysed based on published RNA-seq data (SRA046916) [69]. Clean tags were remapped to the cucumber genome sequence (http://cucurbitgenomics.org/, v3.0) by Biomarker Technologies (Beijing, China), and the FPKM values were recalculated. These analyses were performed on 9 different cucumber tissues: roots, stems, leaves, female flowers, male flowers, ovaries, expanded unfertilized ovaries, expanded fertilized ovaries, and tendrils. Only one biological replication was used for each tissue sample [69]. The genome-wide expression of the *CsWRKY* genes was shown on a heatmap using MeV (Multiple Experiment Viewer) software, and the expression levels are shown by a colour bar that changes from green to red.

### Transcriptome analysis of *CsWRKYs* in response to abiotic and biotic stresses

The expression regulation of *CsWRKY* genes responsive to different stresses was obtained from publicly available transcriptomic data, which were downloaded from Gene Expression Omnibus and analysed to reveal the genome-wide differentially expressed genes after treatment with salt (GSE116265) [70] and inoculation with DM (SRP009350) [54] and PM (GSE81234) [71]. Every treatment had three or two biological replicates. The FDR (or *P* value) and absolute value of log2 (fold-change) that were published in the original literature were used for the identification of DEGs [54, 70, 71]. Because the gene ID shown was according to the cucumber genome v2.0, we cross-referenced the gene IDs of the *CsWRKYs* with those of the cucumber genome v3.0. The expression of the *CsWRKY* genes was then shown by a heatmap using MeV software.

The seedlings of the ‘Chinese long’ inbred line 9930, which was obtained from X. Gu Lab of Institute of Vegetables and Flowers, Chinese Academy of Agricultural Sciences, and used for cucumber genome sequencing, were treated at 42 °C, and the leaves of the seedlings were taken at 0, 3 and 6 h after treatment for transcriptome sequencing in Novogene (Beijing, China). Three biological replicates were performed. The transcript abundance of *CsWRKY* genes was calculated as fragments per kilobase of exon model per million mapped reads (FPKM). The sequencing reads data were submitted to the National Center for Biotechnology Information (NCBI) GEO Sequence Read Archive with accession number of GSE151055.

For the transcriptome analysis of *CsWRKYs* in response to abiotic and biotic stresses, a threshold of FDR (or *P* value) ≤ 0.05 and an absolute value of log2 (fold-change) ≥ 1 were used to define DEGs.

## Supplementary information

**Additional file 1: Figure S1.** The *WRKY* genes we identified mapped on every chromosome according to the current version of cucumber genome (v3.0).

**Additional file 2: Figure S2.** The number of *WRKY* genes mapped on every chromosome according to cucumber genome v1.0 (previous study) and v3.0 (this study).

**Additional file 3: Table S1.** List of the 61 *CsWRKY* genes identified in this study.

**Additional file 4: Table S2.** List of WRKY domains sequence derived from cucumber and Arabidopsis.

**Additional file 5: Table S3.** List of WRKY domain sequences of WRKY IIa proteins.

**Additional file 6: Table S4.** Analysis of conserved motifs in cucumber WRKY proteins.

**Additional file 7: Table S5.** Segmentally duplicated *CsWRKY* gene pairs.

**Additional file 8: Table S6.** Orthologous relationships between cucumber and Arabidopsis/tomato/watermelon/rice/maize.

**Additional file 9: Table S7.** List FPKM of *WRKY* genes in cucumber different tissues.

**Additional file 10: Table S8.** RNA-seq data (FPKM values) of *CsWRKY* genes under salt/heat treatment/powdery mildew /downy mildew infection.

**Additional file 11: Figure S3.** Hierarchical clustering of cucumber gene expression profiles under heat treatment. A, Hierarchical cluster of expressed cucumber genes in 9 samples. In the color panels, each transverse line represents a single gene and the color of the line indicates the expression level of the gene relative to the mean center in a specific sample: red, high expression; green, low expression level. B, The hierarchical clustering on the gene expression matrix, using the Pearson correlation coefficient as a proxy of similarity between transcriptomes. HT0h = heat treatment for 0 h (hours), HT3h = heat treatment for 3 h, HT6h = heat treatment for 6 h.

## Data Availability

The datasets supporting the conclusions of this article are included within the article and its additional files; Cucumber sequences in this article can be found from the CuGenDB (http://cucurbitgenomics.org/); The *Arabidopsis thaliana* sequences in this article were downloaded from TAIR (https://www.arabidopsis.org/index.jsp). The public transcriptome data are available at Gene Expression Omnibus (GEO) (https://www.ncbi.nlm.nih.gov/geo/). The sequencing reads data of cucumber in response to high temperature stress are deposited at GEO with accession number of GSE151055 (https://www.ncbi.nlm.nih.gov/geo/query/acc.cgi?acc=GSE151055). All plant materials were selected from cucumber provided by the Z. Ren lab, Shandong Agricultural University, Taian, China.

## Abbreviations

TFs: Transcription factors
HMM: Hidden Markov Model
CDS: Coding DNA sequence
MW: Molecular weight
pI: Isoelectric point
aa: amino acid
NJ: Neighbor-joining
R protein: Resistance protein
ORF: Open reading frame
DEGs: Differential expression genes
h: Hours
Si: Silicon
DM: Downy mildew
PM: Powdery mildew

## References

[1] Bai Y, Kissoudis C, Yan Z, Visser RGF, van der Linden G (2018). Plant behaviour under combined stress: tomato responses to combined salinity and pathogen stress. Plant J..

[2] Garbeva P, Weisskopf L (2020). Airborne medicine: bacterial volatiles and their influence on plant health. New Phytol..

[3] Kang H, Zhang M, Zhou S, Guo Q, Chen F, Wu J, Wang W (2016). Overexpression of wheat ubiquitin gene, *Ta-Ub2*, improves abiotic stress tolerance of *Brachypodium distachyon*. Plant Sci..

[4] Boyer JS (1982). Plant productivity and environment. Science..

[5] Wang G, Zhang S, Ma X, Wang Y, Kong F, Meng Q (2016). A stress-associated NAC transcription factor (SlNAC35) from tomato plays a positive role in biotic and abiotic stresses. Physiol Plant..

[6] Basso MF, Ferreira PCG, Kobayashi AK, Harmon FG, Nepomuceno AL, Molinari HBC, Grossi-de-Sa MF (2019). MicroRNAs and new biotechnological tools for its modulation and improving stress tolerance in plants. Plant Biotechnol J..

[7] Niklas KJ (2009). Functional adaptation and phenotypic plasticity at the cellular and whole plant level. J Biosci..

[8] Wang G, Xu X, Wang H, Liu Q, Yang X, Liao L, Cai G (2019). A tomato transcription factor, SlDREB3 enhances the tolerance to chilling in transgenic tomato. Plant Physiol Biochem..

[9] Cao ZH, Zhang SZ, Wang RK, Zhang RF, Hao YJ (2013). Genome wide analysis of the apple MYB transcription factor family allows the identification of *MdoMYB121* gene confering abiotic stress tolerance in plants. PLoS One..

[10] Yan H, Jia H, Chen X, Hao L, An H, Guo X (2014). The cotton WRKY transcription factor GhWRKY17 functions in drought and salt stress in transgenic *Nicotiana benthamiana* through ABA signaling and the modulation of reactive oxygen species production. Plant Cell Physiol..

[11] Zhang YL, Zhang CL, Wang GL, Wang YX, Qi CH, You CX, Li YY, Hao YJ (2019). Apple AP2/EREBP transcription factor MdSHINE2 confers drought resistance by regulating wax biosynthesis. Planta..

[12] Le Henanff G, Profizi C, Courteaux B, Rabenoelina F, Gerard C, Clement C, Baillieul F, Cordelier S, Dhondt-Cordelier S (2013). Grapevine NAC1 transcription factor as a convergent node in developmental processes, abiotic stresses, and necrotrophic/biotrophic pathogen tolerance. J Exp Bot..

[13] Liu B, Ouyang Z, Zhang Y, Li X, Hong Y, Huang L, Liu S, Zhang H, Li D, Song F (2014). Tomato NAC transcription factor SlSRN1 positively regulates defense response against biotic stress but negatively regulates abiotic stress response. PLoS One..

[14] Riechmann JL, Ratcliffe OJ (2000). A genomic perspective on plant transcription factors. Curr Opin Plant Biol..

[15] Ulker B, Somssich IE (2004). WRKY transcription factors: from DNA binding towards biological function. Curr Opin Plant Biol..

[16] Dong J, Chen C, Chen Z (2003). Expression profiles of the *Arabidopsis WRKY* gene superfamily during plant defense response. Plant Mol Biol..

[17] Ross CA, Liu Y, Shen QJ (2007). The *WRKY* gene family in rice (*Oryza sativa*). J Integr Plant Biol..

[18] Zhang T, Tan D, Zhang L, Zhang X, Han Z (2017). Phylogenetic analysis and drought-responsive expression profiles of the WRKY transcription factor family in maize. Agri Gene..

[19] Huang S, Gao Y, Liu J, Peng X, Niu X, Fei Z, Cao S, Liu Y (2012). Genome-wide analysis of WRKY transcription factors in *Solanum lycopersicum*. Mol Genet Genomics..

[20] Rinerson CI, Rabara RC, Tripathi P, Shen QJ, Rushton PJ (2015). The evolution of WRKY transcription factors. BMC Plant Biol..

[21] An JP, Zhang XW, You CX, Bi SQ, Wang XF, Hao YJ (2019). MdWRKY40 promotes wounding-induced anthocyanin biosynthesis in association with MdMYB1 and undergoes MdBT2-mediated degradation. New Phytol..

[22] Xie T, Chen C, Li C, Liu J, Liu C, He Y (2018). Genome-wide investigation of *WRKY* gene family in pineapple: evolution and expression profiles during development and stress. BMC Genomics..

[23] Wu KL, Guo ZJ, Wang HH, Li J (2005). The WRKY family of transcription factors in rice and *Arabidopsis* and their origins. DNA Res..

[24] Zhang Y, Wang L (2005). The WRKY transcription factor superfamily: its origin in eukaryotes and expansion in plants. BMC Evol Biol..

[25] Eulgem T, Rushton PJ, Robatzek S, Somssich IE (2000). The WRKY superfamily of plant transcription factors. Trends Plant Sci..

[26] Rushton PJ, Somssich IE, Ringler P, Shen QJ (2010). WRKY transcription factors. Trends Plant Sci..

[27] Gu L, Dou L, Guo Y, Wang H, Li L, Wang C, Ma L, Wei H, Yu S (2019). The WRKY transcription factor *GhWRKY27* coordinates the senescence regulatory pathway in upland cotton (*Gossypium hirsutum* L.). BMC Plant Biol..

[28] Zhang S, Li C, Wang R, Chen Y, Shu S, Huang R, Zhang D, Li J, Xiao S, Yao N (2017). The Arabidopsis Mitochondrial Protease FtSH4 Is Involved in Leaf Senescence via Regulation of WRKY-Dependent Salicylic Acid Accumulation and Signaling. Plant Physiol..

[29] Pesch M, Dartan B, Birkenbihl R, Somssich IE, Hulskamp M (2014). *Arabidopsis* TTG2 regulates *TRY* expression through enhancement of activator complex-triggered activation. Plant Cell..

[30] Duan S, Wang J, Gao C, Jin C, Li D, Peng D, Du G, Li Y, Chen M (2018). Functional characterization of a heterologously expressed *Brassica napus WRKY41-1* transcription factor in regulating anthocyanin biosynthesis in Arabidopsis thaliana. Plant Sci..

[31] Gonzalez A, Brown M, Hatlestad G, Akhavan N, Smith T, Hembd A, Moore J, Montes D, Mosley T, Resendez J (2016). TTG2 controls the developmental regulation of seed coat tannins in *Arabidopsis* by regulating vacuolar transport steps in the proanthocyanidin pathway. Dev Biol..

[32] Chen M, Yan T, Shen Q, Lu X, Pan Q, Huang Y, Tang Y, Fu X, Liu M, Jiang W (2017). *GLANDULAR TRICHOME-SPECIFIC WRKY 1* promotes artemisinin biosynthesis in *Artemisia annua*. New Phytol..

[33] Yu Y, Liu Z, Wang L, Kim SG, Seo PJ, Qiao M, Wang N, Li S, Cao X, Park CM (2016). WRKY71 accelerates flowering via the direct activation of *FLOWERING LOCUS T* and *LEAFY* in *Arabidopsis thaliana*. Plant J..

[34] Zhang L, Chen L, Yu D (2018). Transcription Factor WRKY75 Interacts with DELLA Proteins to Affect Flowering. Plant Physiol..

[35] Johnson CS, Kolevski B, Smyth DR (2002). *TRANSPARENT TESTA GLABRA2*, a trichome and seed coat development gene of Arabidopsis, encodes a WRKY transcription factor. Plant Cell..

[36] Jiang W, Yu D (2009). *Arabidopsis WRKY2* transcription factor mediates seed germination and postgermination arrest of development by abscisic acid. BMC Plant Biol..

[37] Ding ZJ, Yan JY, Li GX, Wu ZC, Zhang SQ, Zheng SJ (2014). WRKY41 controls Arabidopsis seed dormancy via direct regulation of *ABI3* transcript levels not downstream of ABA. Plant J..

[38] Jiang Y, Deyholos MK (2006). Comprehensive transcriptional profiling of NaCl-stressed Arabidopsis roots reveals novel classes of responsive genes. BMC Plant Biol..

[39] Chen YF, Li LQ, Xu Q, Kong YH, Wang H, Wu WH (2009). The WRKY6 transcription factor modulates *PHOSPHATE1* expression in response to low Pi stress in *Arabidopsis*. Plant Cell..

[40] Kim KC, Lai Z, Fan B, Chen Z (2008). *Arabidopsis* WRKY38 and WRKY62 transcription factors interact with histone deacetylase 19 in basal defense. Plant Cell..

[41] Lai Z, Vinod K, Zheng Z, Fan B, Chen Z (2008). Roles of *Arabidopsis* WRKY3 and WRKY4 transcription factors in plant responses to pathogens. BMC Plant Biol..

[42] Chen X, Liu J, Lin G, Wang A, Wang Z, Lu G (2013). Overexpression of *AtWRKY28* and *AtWRKY75* in *Arabidopsis* enhances resistance to oxalic acid and *Sclerotinia sclerotiorum*. Plant Cell Rep..

[43] Pandey SP, Roccaro M, Schon M, Logemann E, Somssich IE (2010). Transcriptional reprogramming regulated by WRKY18 and WRKY40 facilitates powdery mildew infection of Arabidopsis. Plant J..

[44] Jammes F, Lecomte P (2005). de Almeida-Engler J, Bitton F, Martin-Magniette ML, Renou JP, Abad P, Favery B. Genome-wide expression profiling of the host response to root-knot nematode infection in Arabidopsis. Plant J..

[45] Grunewald W, Karimi M, Wieczorek K, Van de Cappelle E, Wischnitzki E, Grundler F, Inze D, Beeckman T, Gheysen G (2008). A role for AtWRKY23 in feeding site establishment of plant-parasitic nematodes. Plant Physiol..

[46] Bai Y, Sunarti S, Kissoudis C, Visser RGF, van der Linden CG (2018). The Role of Tomato *WRKY* Genes in Plant Responses to Combined Abiotic and Biotic Stresses. Front Plant Sci..

[47] He Y, Mao S, Gao Y, Zhu L, Wu D, Cui Y, Li J, Qian W (2016). Genome-Wide Identification and Expression Analysis of WRKY Transcription Factors under Multiple Stresses in *Brassica napus*. PLoS One..

[48] Yu Y, Wang N, Hu R, Xiang F (2016). Genome-wide identification of soybean WRKY transcription factors in response to salt stress. Springerplus..

[49] Jimmy JL, Babu S (2015). Role of OsWRKY transcription factors in rice disease resistance. Trop Plant Pathol..

[50] Chen L, Zhao Y, Xu S, Zhang Z, Xu Y, Zhang J, Chong K (2018). OsMADS57 together with OsTB1 coordinates transcription of its target *OsWRKY94* and *D14* to switch its organogenesis to defense for cold adaptation in rice. New Phytol..

[51] Okay S, Derelli E, Unver T (2014). Transcriptome-wide identification of bread wheat WRKY transcription factors in response to drought stress. Mol Genet Genomics..

[52] Huang S, Li R, Zhang Z, Li L, Gu X, Fan W, Lucas WJ, Wang X, Xie B, Ni P (2009). The genome of the cucumber, *Cucumis sativus* L. Nat Genet..

[53] Luan Q, Chen C, Liu M, Li Q, Wang L, Ren Z (2019). *CsWRKY50* mediates defense responses to *Pseudoperonospora cubensis* infection in Cucumis sativus. Plant Sci..

[54] Adhikari BN, Savory EA, Vaillancourt B, Childs KL, Hamilton JP, Day B, Buell CR (2012). Expression profiling of *Cucumis sativus* in response to infection by *Pseudoperonospora cubensis*. PLoS One..

[55] Ling J, Jiang W, Zhang Y, Yu H, Mao Z, Gu X, Huang S, Xie B (2011). Genome-wide analysis of WRKY gene family in *Cucumis sativus*. BMC Genomics..

[56] Li Q, Li H, Huang W, Xu Y, Zhou Q, Wang S, Ruan J, Huang S, Zhang Z. A chromosome-scale genome assembly of cucumber (*Cucumis sativus* L.). Gigascience. 2019;8(6). 10.1093/gigascience/giz072 PMC6582320 31216035

[57] Tripathi P, Rabara RC, Langum TJ, Boken AK, Rushton DL, Boomsma DD, Rinerson CI, Rabara J, Reese RN, Chen X (2012). The WRKY transcription factor family in *Brachypodium distachyon*. BMC Genomics..

[58] Ding M, Chen J, Jiang Y, Lin L, Cao Y, Wang M, Zhang Y, Rong J, Ye W (2014). Genome-wide investigation and transcriptome analysis of the *WRKY* gene family in *Gossypium*. Mol Genet Genomics..

[59] Mangelsen E, Kilian J, Berendzen KW, Kolukisaoglu üH, Harter K, Jansson C, Wanke D (2008). Phylogenetic and comparative gene expression analysis of barley (*Hordeum vulgare*) WRKY transcription factor family reveals putatively retained functions between monocots and dicots. BMC Genomics..

[60] Wei Y, Haitao S, Zhiqiang X, Weiwei T, Zehong D, Yan Y, Wenquan W, Wei H, Kaimian L. Genome-Wide Identification and Expression Analysis of the *WRKY* Gene Family in *Cassava*. Front Plant Sci. 2016;7. 10.3389/fpls.2016.00025 PMC4742560 26904033

[61] Raja K, Sriram V, Backiyarani S, Uma S, Saraswathi MS, Mayilvaganan M (2016). Evolutionary Expansion of *WRKY* Gene Family in Banana and Its Expression Profile during the Infection of Root Lesion Nematode, *Pratylenchus coffeae*. Plos One..

[62] He H, Dong Q, Shao Y, Jiang H, Xiang Y (2012). Genome-wide survey and characterization of the *WRKY* gene family in *Populus trichocarpa*. Plant Cell Rep..

[63] Huang X, Li K, Xu X, Yao Z, Jin C, Zhang S (2015). Genome-wide analysis of WRKY transcription factors in white pear (*Pyrus bretschneideri*) reveals evolution and patterns under drought stress. BMC Genomics..

[64] Li HL, Zhang LB, Guo D, Li CZ, Peng SQ (2012). Identification and expression profiles of the WRKY transcription factor family in *Ricinus communis*. Gene..

[65] Li D, Liu P, Yu J, Wang L, Dossa K, Zhang Y, Zhou R, Wei X, Zhang X (2017). Genome-wide analysis of *WRKY* gene family in the sesame genome and identification of the *WRKY* genes involved in responses to abiotic stresses. BMC Plant Biol..

[66] Liu QN, Liu Y, Xin Z-Z, Zhang D-Z, Ge B-M, Yang R-P, Wang Z-F, Yang L, Tang B-P, Zhou C-L (2017). Genome-wide identification and characterization of the *WRKY* gene family in potato (*Solanum tuberosum*). Biochem Syst Ecol..

[67] Zhang Y, Feng JC (2014). Identification and Characterization of the Grape WRKY Family. Biomed Res Int..

[68] Hu R, Qi G, Kong Y, Kong D, Gao Q, Zhou G (2010). Comprehensive analysis of NAC domain transcription factor gene family in *Populus trichocarpa*. BMC Plant Biol..

[69] Li Z, Zhang Z, Yan P, Huang S, Lin K (2011). RNA-Seq improves annotation of protein-coding genes in the cucumber genome. BMC Genomics..

[70] Zhu Y, Yin J, Liang Y, Liu J, Jia J (2019). Transcriptomic dynamics provide an insight into the mechanism for silicon-mediated alleviation of salt stress in cucumber plants. Ecotoxicol Environ Saf..

[71] Xu Q, Xu X, Shi Y, Qi X, Chen X (2017). Elucidation of the molecular responses of a cucumber segment substitution line carrying *Pm5.1* and its recurrent parent triggered by powdery mildew by comparative transcriptome profiling. BMC Genomics..

[72] Debona D, Rodrigues FA, Datnoff LE (2017). Silicon's Role in Abiotic and Biotic Plant Stresses. Annu Rev Phytopathol..

[73] Brand LH, Fischer NM, Harter K, Kohlbacher O, Wanke D (2013). Elucidating the evolutionary conserved DNA-binding specificities of WRKY transcription factors by molecular dynamics and in vitro binding assays. Nucleic Acids Res..

[74] Wei KF, Chen J, Chen YF, Wu LJ, Xie DX (2012). Molecular phylogenetic and expression analysis of the complete WRKY transcription factor family in maize. DNA Res..

[75] Xu Z, Sun L, Zhou Y, Yang W, Cheng T, Wang J, Zhang Q (2015). Identification and expression analysis of the SQUAMOSA promoter-binding protein (SBP)-box gene family in *Prunus mume*. Mol Genet Genomics..

[76] Grunewald W, Smet ID, Lewis DR, Löfke C, Beeckman T (2012). Transcription factor WRKY23 assists auxin distribution patterns during *Arabidopsis* root development through local control on flavonol biosynthesis. Proc Natl Acad Sci U S A..

[77] Stetter MG, Benz M, Ludewig U (2017). Increased root hair density by loss of *WRKY6* in *Arabidopsis thaliana*. Peerj..

[78] Ramamoorthy R, Jiang SY, Kumar N, Venkatesh PN, Ramachandran S (2008). A comprehensive transcriptional profiling of the *WRKY* gene family in rice under various abiotic and phytohormone treatments. Plant Cell Physiol..

[79] Scarpeci TE, Zanor MI, Mueller-Roeber B, Valle EM (2013). Overexpression of *AtWRKY30* enhances abiotic stress tolerance during early growth stages in *Arabidopsis thaliana*. Plant Mol Biol..

[80] Liu Q, Li X, Yan S, Yu T, Yang J, Dong J, Zhang S, Zhao J, Yang T, Mao X. OsWRKY67 positively regulates blast and bacteria blight resistance by direct activation of *PR* genes in rice. BMC Plant Biol. 2018;18(1). 10.1186/s12870-018-1479-y PMC6204034 30367631

[81] Muthamilarasan M, Bonthala VS, Khandelwal R, Jaishankar J, Shweta S, Nawaz K, Prasad M (2015). Global analysis of WRKY transcription factor superfamily in Setaria identifies potential candidates involved in abiotic stress signaling. Front Plant Sci..

[82] Wang Y, Tang H, DeBarry JD, Tan X, Li J, Wang X, Lee T-h, Jin H, Marler B, Guo H (2012). *MCScanX*: a toolkit for detection and evolutionary analysis of gene synteny and collinearity. Nucleic Acids Res..

