# Supplementary material for: Genome-wide analysis of the WRKY gene family in the cucumber genome and transcriptome-wide identification of WRKY transcription factors that respond to biotic and abiotic stresses
Source: BMC Plant Biol. 2020 Sep 25;20:443. doi: 10.1186/s12870-020-02625-8 (PMC7517658; doi:10.1186/s12870-020-02625-8)
Supplement: Supplementary file 1 — Additional file 1: Figure S1. The WRKY genes we identified mapped on every chromosome according to the current version of cucumber genome (v3.0). [file 12870_2020_2625_MOESM1_ESM.pptx]

## Slide 1
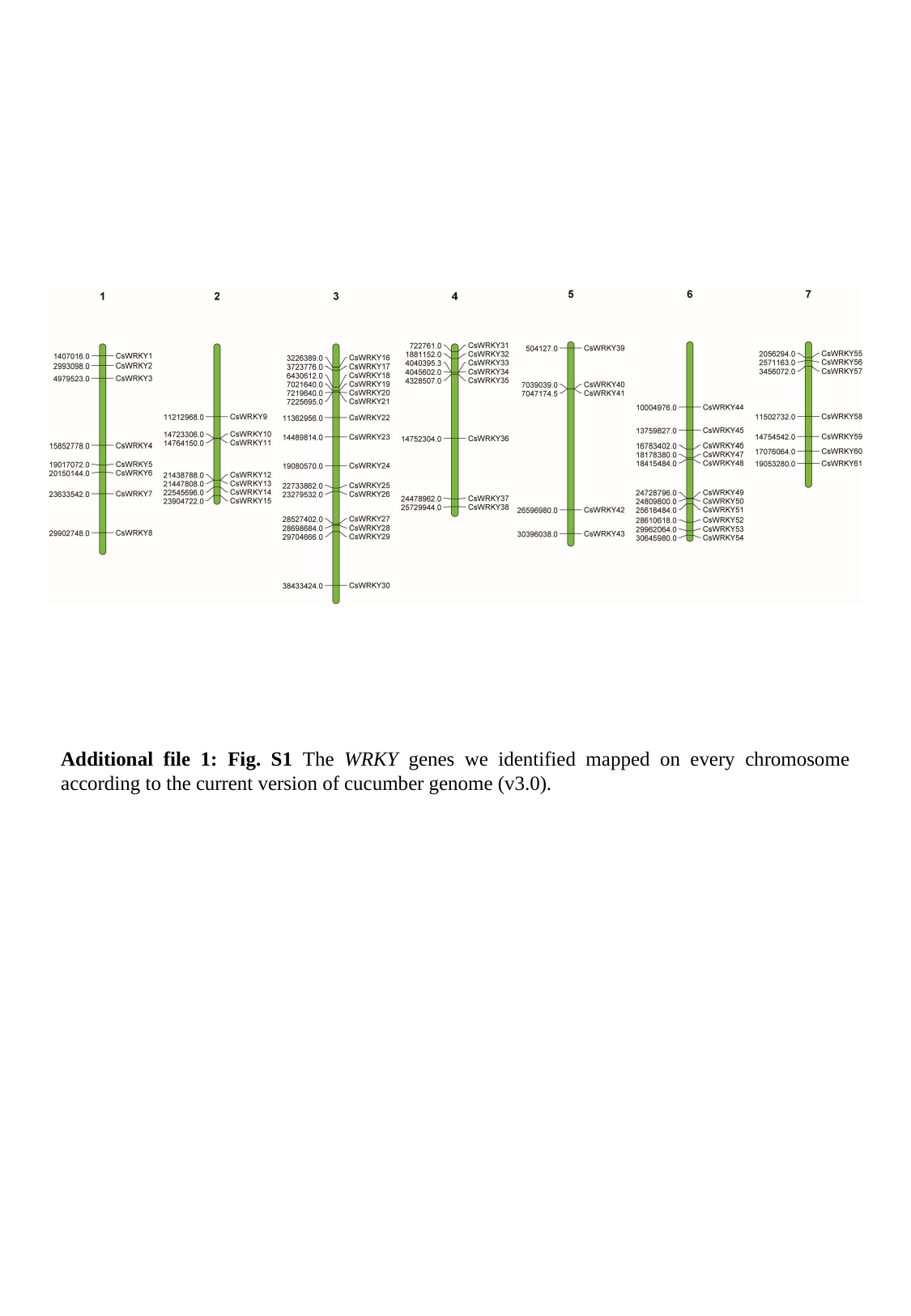

Additional file 1: Fig. S1 The WRKY genes we identified mapped on every chromosome according to the current version of cucumber genome (v3.0).
