# Supplementary material for: Genome-wide analysis of the WRKY gene family in the cucumber genome and transcriptome-wide identification of WRKY transcription factors that respond to biotic and abiotic stresses
Source: BMC Plant Biol. 2020 Sep 25;20:443. doi: 10.1186/s12870-020-02625-8 (PMC7517658; doi:10.1186/s12870-020-02625-8)
Supplement: Supplementary file 2 — Additional file 2: Figure S2. The number of WRKY genes mapped on every chromosome according to cucumber genome v1.0 (previous study) and v3.0 (this study). [file 12870_2020_2625_MOESM2_ESM.pptx]

## Slide 1
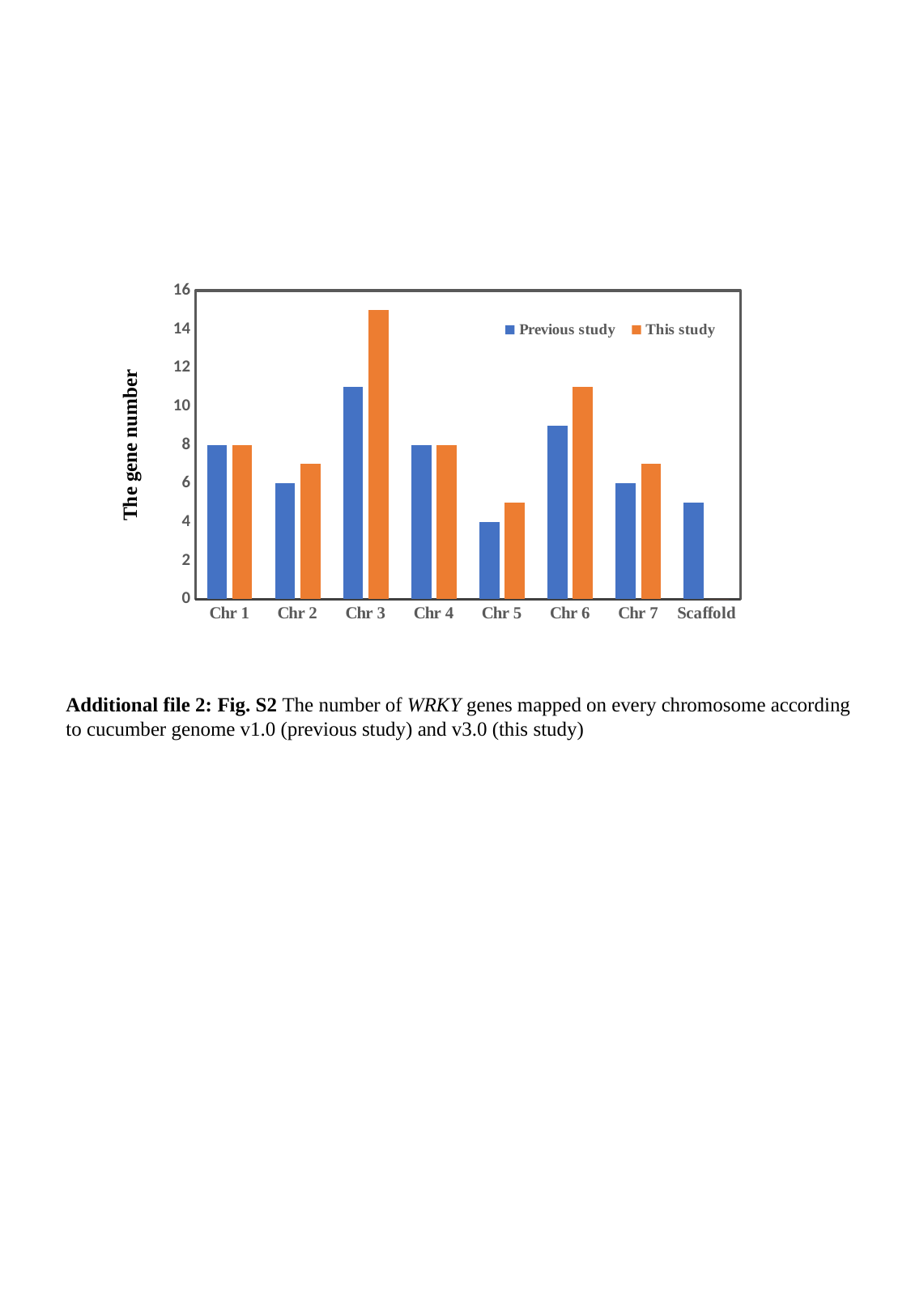

The gene number
### Chart
| Category | Previous study | This study |
|---|---|---|
| Chr 1 | 8.0 | 8.0 |
| Chr 2 | 6.0 | 7.0 |
| Chr 3 | 11.0 | 15.0 |
| Chr 4 | 8.0 | 8.0 |
| Chr 5 | 4.0 | 5.0 |
| Chr 6 | 9.0 | 11.0 |
| Chr 7 | 6.0 | 7.0 |
| Scaffold | 5.0 | 0.0 |Additional file 2: Fig. S2 The number of WRKY genes mapped on every chromosome according to cucumber genome v1.0 (previous study) and v3.0 (this study)
