# Supplementary material for: Genome-wide analysis of the WRKY gene family in the cucumber genome and transcriptome-wide identification of WRKY transcription factors that respond to biotic and abiotic stresses
Source: BMC Plant Biol. 2020 Sep 25;20:443. doi: 10.1186/s12870-020-02625-8 (PMC7517658; doi:10.1186/s12870-020-02625-8)
Supplement: Supplementary file 4 — Additional file 4: Table S2. List of WRKY domains sequence derived from cucumber and Arabidopsis. [file 12870_2020_2625_MOESM4_ESM.docx]

**Additional file 4: Table S2** List of WRKY domains sequence derived from cucumber and Arabidopsis.

| **Gene Name** | **Sequence Type** | **Sequences** |
| --- | --- | --- |
| CsWRKY1 | WRKY domain | NDGCQWRKYGQKIAKGNPCPRAYYRCTGAPTCPVRKQVQRSVDDISILITTYEGTHNHP |
| CsWRKY2 | WRKY domain-N | DDGYNWRKYGQKQVKGSENPRSYYKCTFPNCPTKKKVERSLDGQITEIVYKGSHNHP |
|  | WRKY domain-C | DDGYRWRKYGQKVVKGNPNPRSYYKCTNPGCPVRKHVERASHDLRAVITTYEGKHNHD |
| CsWRKY3 | WRKY domain | NDGCQWRKYGQKIAKGNPCPRAYYRCTGSPTCPVRKQVQRCADDMSILITTYEGNHNHP |
| CsWRKY4 | WRKY domain-N | DGYNWRKYGQKQVKSPKGSRSYYKCTYSECFAKKIECCDDSGQTTEIVYKSQHSHD |
|  | WRKY domain-C | DGYRWRKYGQKMVKGNPHPRNYYRCTSAGCPVRKHIESAVENPNAVIITYKGVHDHD |
| CsWRKY5 | WRKY domain | DEYSWRKYGQKPIKGSPYPRGYYRCSSVKGCPARKKVERARDDPAMLLVTYEGDHRHP |
| CsWRKY6 | WRKY domain | DSWAWRKYGQKPIKGSPYPRAYYRCSSSKGCPARKQVERNRLDPTTLVITYSCEHNH |
| CsWRKY7 | WRKY domain | DLWAWRKYGQKPIKGSPYPRGYYRCSSSKGCsRKQVERSRTNPNMLVITYTSEHNHP |
| CsWRKY8 | WRKY domain-N | EDGYNWRKYGQKQVKGSEYPRSYYKCTHPNCLVKKKVERSLDGQITEIIYKGAHNH |
|  | WRKY domain-C | EDGYRWRKYGQKVVKGNPNPRSYYKCTSAGCLVRKHVERASHDLKCVITTYEGKHNHE |
| CsWRKY9 | WRKY domain | DDYSWRKYGQKPIKGSPHPRGYYKCSSIRGCPARKHVERCLEDPSMLIVTYEGEHNHP |
| CsWRKY10 | WRKY domain | DDGYKWRKYGKKSVKNSPNPRNYYKCSSEGCNVKKKVERDREDANYVITTYEGIHNHE |
| CsWRKY11 | WRKY domain | DDGYKWRKYGQKVVKNTQHPRSYYRCTQDHCRVKKRVERLAEDPRMVITTYEGRHVH |
| CsWRKY12 | WRKY domain | DGYQWRKYGQKVTKDNPSPRAYYKCSFAPTCPVKRKVQRSVEEPCYLVATYEGQHNHP |
| CsWRKY13 | WRKY domain | DGYKWRKYGQKITKDNQSPRAYFKCSSPGCPVKKKVQRSLENKSMVIVTYDGHHNHN |
| CsWRKY14 | WRKY domain | DDGYRWRKYGQKAVKNNKFPRSYYKCSNEGCKVKKQIQRLTN |
| CsWRKY15 | WRKY domain | DDYSWRKYGQKPIKGSPYPRGYYKCSSLRGCPARKHVERASDDPSMLIVTYEGDHNH |
| CsWRKY16 | WRKY domain-N | EDGYNWRKYGQKQVKGSEYPRSYYKCTHPNCQVKKKVERSHEGHITEIIYKGTHNHP |
|  | WRKY domain-C | DDGYRWRKYGQKVVKGNPNPRSYYKCTNPGCTVRKHVERASHDLKSVITTYEGKHNHD |
| CsWRKY17 | WRKY domain-N | DDGYNWRKYGQKHVKGSEFPRSYYKCTHPNCEVKKLFERSHDGQITDIIYKGTHDHP |
|  | WRKY domain-C | DDGYRWRKYGQKVVRGNPNPRSYYKCTNVGCPVRKHVERASHDPKAVITTYEGKHNHD |
| CsWRKY18 | WRKY domain | DDGYKWRKYGQKVVKNSLHPRSYYRCTHSNCRVKKRVERLSEDCRMVITTYEGRHNH |
| CsWRKY19 | WRKY domain | NDGYSWRKYGQKDIHGANFPRCYYRCTHRNVRGCLATKQVQKSDNDPNIFEVTYRGRHT |
| CsWRKY20 | WRKY domain | DDGYRWRKYGQKAVKHSLHPRSYYKCTYVTCNVKKQVQRLSKDRSIVVTTYEGIHNHP |
| CsWRKY21 | WRKY domain | DIWAWRKYGQKPIKGSPYPRGYYRCSSSKGCMARKQVERNRSDPGMFIVTYTAEHNHP |
| CsWRKY22 | WRKY domain | DDGYRWRKYGQKAVKNSPYPRSYYRCTTAGCGVKKRVERSSGDHTIVVTTYEGQHTHQ |
| CsWRKY23 | WRKY domain | NDGCQWRKYGQKIAKGNPCPRAYYRCTVAPGCPVRKQVQRCLEDMSILITTYEGTHNHP |
| CsWRKY24 | WRKY domain | DDGFAWRKYGQKGILGAKHPRGYYRCTHRNLQGCLATKQVQRSDDDPTIFEITYRGKHS |
| CsWRKY25 | WRKY domain | EDGYRWRKYGQKAVKNSPFPRSYYRCTSAACNVKKRVERSFADPTVVVTTYEGQHTHP |
| CsWRKY26 | WRKY domain | DDGYRWRKYGQKAVKNNKFPRSYYRCTHQGCNVKKQVQRLTRDEGVVVTTYEGMHTH |
| CsWRKY27 | WRKY domain | DDGHAWRKYGQKTILNAKYPRNYYRCTHKYDQTCQATKQVQRLQDNPPKFRTTYYGNH |
| CsWRKY28 | WRKY domain-N | NDGYNWRKYGQKQVKGSENPRSYYKCTFPSCPTKKKVERSLDGQITEIVYKGTHNH |
|  | WRKY domain-C | DGYRWRKYGQKVVKGNPNPRSYYKCTSLGCPVRKHIERAANDMRAVITTYEGKHNHE |
| CsWRKY29 | WRKY domain-N | DGYNWRKYGQKQVKGSEYPRSYYKCTHPSCPVKKKVERSLDGKVAEIVYKGEHNHP |
|  | WRKY domain-C | GVRWRKYGQKVVKGNLYPRSYYRCTGLKCKARKYVERASEDPDSFITTYEGKHNH |
| CsWRKY30 | WRKY domain | DEYSWRKYGQKPIKGSPYPRGYYKCSTMRGCPARKHVERDPNDPAMLIVTYEGEHRH |
| CsWRKY31 | WRKY domain | DFWSWRKYGQKPIKGSPYPRGYYRCSTTKGCsKKQVERCKTDGSMFIITYTSSHNHP |
| CsWRKY32 | WRKY domain | DDGYRWRKYGQKAVKNNKFPRSYYRCTHQGCKVKKQVQRLTRDEGVVVTTYEGIHSHP |
| CsWRKY33 | WRKY domain | DSWGWRKYGQKPIKGSPYPRSYYRCSSSKGCsRKQVERSLSDPEVFIVTYTAEHNH |
| CsWRKY34 | WRKY domain | DDGYRWRKYGQKAVKHSNHPRSYYRCTHHTCNVKKQIQRHSKDPTIVVTTYEGIHNHP |
| CsWRKY35 | WRKY domain | EDGYSWRKYGQKDILGATYPRSYYRCTFRNTQNCWAVKQVQRSDEDPSVFEITYRGKHT |
| CsWRKY36 | WRKY domain | DDGYKWRKYGQKSIKNSPNPRSYYRCSNPRCSAKKQVERSIEDPDIFIITYEGLHLHFA |
| CsWRKY37 | WRKY domain | DDYSWRKYGQKPIKGSPHPRGYYKCSSLRGCPARKHVERALDDPTMLIVTYENDHNH |
| CsWRKY38 | WRKY domain | DGYQWRKYGQKVTRDNPSPRAYFKCSSAPNCPVKKKVQRSLEDPTILVATYEGEHSH |
| CsWRKY39 | WRKY domain | DMWAWRKYGQKPIKGSPYPRNYYRCSSSKGCGARKQVERSNDDPETFTITYTGDHSHP |
| CsWRKY40 | WRKY domain | PDDGFTWRKYGQKEILGSRFPRFVFRFFYFPLFSFHTFHFL |
| CsWRKY41 | WRKY domain | EDNYGWRKYGQKAIHNTTYPRSYYRCTHKFDQGCQATKQVQRMEGDDSEIMYN |
| CsWRKY42 | WRKY domain | DDGYNWRKYGQKQVKGSEFPRSYYKCTHPNCPVKKKVERSLEGQVTEIIYKGEHNH |
| CsWRKY43 | WRKY domain-N | DDGYNWRKYGQKLVKGSEFPRSYYKCTHLNCPVKKKIERSPDGQITEIIYKGQHNHE |
|  | WRKY domain-C | DDGYRWRKYGQKVVKGNPNPRSYYKCTSAGCNVRKHVERSSTDSKAVVTTYEGKHNHD |
| CsWRKY44 | WRKY domain | EDGYRWRKYGQKAVKNSPFPRSYYKCTSQNCSVKKRVERSSEDPSFVITTYEGKHNH |
| CsWRKY45 | WRKY domain-N | EDGFNWRKYGQKVVKGSENPRSYYKCTFPNCPVRKQVERSLNNNGQITEIVYKSKHNHP |
|  | WRKY domain-C | DDGYWWRKYGQKVVKGNPNPRSYYKCTYPGCGVRKHIERASHDFRAVVTTYEGKHNHD |
| CsWRKY46 | WRKY domain | DDGYRWRKYGQKAVKNSPYPRSYYRCTTAGCGVKKRVERSSDDPSIVVTTYEGQHTHQ |
| CsWRKY47 | WRKY domain | DDGFKWRKYGKKMVKNSPNPRNYYKCSVEGCPVKKRVERDR |
| CsWRKY48 | WRKY domain | EDGYRWRKYGQKAVKNSPFPRSYYRCTNSKCTVKKRVERSCEDSSVVITTYEGQHCH |
| CsWRKY49 | WRKY domain | DDGYKWRKYGQKVVKNTLHPRSYYRCTEENCKVKKRVERLADDPRMVITTYEGRHAH |
| CsWRKY50 | WRKY domain | DDGFKWRKYGKKSVKNSPHPRNYYKCSSGECGVKKRVERDRDDSSYVITTYEGVHNHE |
| CsWRKY51 | WRKY domain | DDYSWRKYGQKPIKGSPHPRGYYKCSSVRGCPARKHVERAVDDPAMLVVTYEGEHNH |
| CsWRKY52 | WRKY domain | DGCQWRKYGQKMAKGNPCPRAYYRCTMAVGCPVRKQVQRCAEDRTILITTYEGNHNHP |
| CsWRKY53 | WRKY domain | EDGYRWRKYGQKAVKNSAYPRSYYRCTTQKCGVKKRVERSYEDPSIVITTYEGQHNH |
| CsWRKY54 | WRKY domain | DLWAWRKYGQKPIKGSPYPRGYYRCSSSKGCsRKQVERSRTDPNMLVITYTSEHNHP |
| CsWRKY55 | WRKY domain-N | EDGFNWRKYGQKLVKGNVFVRSYYRCTHPTCMVKKQLERTHDGKITDTVYFGQHDHP |
|  | WRKY domain-C | NDGYRWRKYGQKFVKGNPNPRSYYRCSSPGCPVKKHVERASHDPKIVLTTYEGQHDH |
| CsWRKY56 | WRKY domain | DGCQWRKYGQKMAKGNPCPRAYYRCTMALGCPVRKQVQRCAEDKTILITTYEGNHNHP |
| CsWRKY57 | WRKY domain | DSWAWRKYGQKPIKGSPYPRGYYRCSSSKGCPARKQVERSRVDPTKLVITYAFDHNHQ |
| CsWRKY58 | WRKY domain | EDGYRWRKYGQKAVKNSPYPRSYYRCTSQKCVVKKRVERSYQDPSVVITTYEGQHNH |
| CsWRKY59 | WRKY domain | DGFSWRKYGQKDILGSKFPRSYFRCSHRFTQGCLATKQVQKSDNDPTIYEVTYKGRHT |
| CsWRKY60 | WRKY domain | DDYSWRKYGQKPIKGSPHPRGYYKCSSMRGCPARKHVERCLEEPSMLIVTYEGEHNHP |
| CsWRKY61 | WRKY domain | EDGYRWRKYGQKAVKNSPHPRSYYRCTSVACNVKKRVERCLQDPSIVVTTYEGQHTHP |
| AtWRKY1 | WRKY domain-N | EDGYNWRKYGQKLVKGNEFVRSYYRCTHPNCKAKKQLERSAGGQVVDTVYFGEHDHP |
| AtWRKY2 | WRKY domain-N | EDGYNWRKYGQKLVKGSEYPRSYYKCTNPNCQVKKKVERSREGHITEIIYKGAHNH |
| AtWRKY3 | WRKY domain-N | DDGYNWRKYGQKQVKGSDFPRSYYKCTHPACPVKKKVERSLDGQVTEIIYKGQHNHE |
| AtWRKY4 | WRKY domain-N | DDGYNWRKYGQKQVKGSEFPRSYYKCTNPGCPVKKKVERSLDGQVTEIIYKGQHNHE |
| AtWRKY6 | WRKY domain | DGCQWRKYGQKMAKGNPCPRAYYRCTMATGCPVRKQVQRCAEDRSILITTYEGNHNHP |
| AtWRKY7 | WRKY domain | DEFSWRKYGQKPIKGSPHPRGYYKCSSVRGCPARKHVERALDDAMMLIVTYEGDHNH |
| AtWRKY8 | WRKY domain | EDGYRWRKYGQKAVKNSPYPRSYYRCTTQKCNVKKRVERSYQDPTVVITTYESQHNHP |
| AtWRKY9 | WRKY domain | NDGCQWRKYGQKTAKGNPCPRAYYRCTVAPGCPVRKQVQRCLEDMSILITTYEGTHNHP |
| AtWRKY10 | WRKY domain | NDGYRWRKYGQKVVKGNPNPRSYFKCTNIECRVKKHVERGADNIKLVVTTYDGIHNHP |
| AtWRKY11 | WRKY domain | DEYSWRKYGQKPIKGSPHPRGYYKCSTFRGCPARKHVERALDDPAMLIVTYEGEHRH |
| AtWRKY12 | WRKY domain | DDGYKWRKYGQKVVKNSLHPRSYYRCTHNNCRVKKRVERLSEDCRMVITTYEGRHNH |
| AtWRKY13 | WRKY domain | DDGYRWRKYGQKVVKNTQHPRSYYRCTQDKCRVKKRVERLADDPRMVITTYEGRHLH |
| AtWRKY14 | WRKY domain | DLWAWRKYGQKPIKGSPFPRGYYRCSSSKGCSARKQVERSRTDPNMLVITYTSEHNHP |
| AtWRKY15 | WRKY domain | DDYSWRKYGQKPIKGSPHPRGYYKCSSVRGCPARKHVERAADDSSMLIVTYEGDHNH |
| AtWRKY16 | WRKY domain | DLWVWRKYGQKPIKSSPYPRSYYRCASSKGCFARKQVERSRTDPNVSVITYISEHNHP |
| AtWRKY17 | WRKY domain | DEYSWRKYGQKPIKGSPHPRGYYKCSTFRGCPARKHVERALDDSTMLIVTYEGEHRH |
| AtWRKY18 | WRKY domain | KDGFQWRKYGQKVTRDNPSPRAYFRCSFAPSCPVKKKVQRSAEDPSLLVATYEGTHNH |
| AtWRKY19 | WRKY domain-N | NDGYNWQKYGQKKVKGSKFPLSYYKCTYLGCPSKRKVERSLDGQVAEIVYKDRHNHE |
| AtWRKY20 | WRKY domain-N | DDGYNWRKYGQKHVKGSEFPRSYYKCTHPNCEVKKLFERSHDGQITDIIYKGTHDHP |
| AtWRKY21 | WRKY domain | DDYSWRKYGQKPIKGSPYPRGYYKCSSMRGCPARKHVERCLEDPAMLIVTYEAEHNHP |
| AtWRKY22 | WRKY domain | DVWAWRKYGQKPIKGSPYPRGYYRCSTSKGCLARKQVERNRSDPKMFIVTYTAEHNHP |
| AtWRKY23 | WRKY domain | EDGYRWRKYGQKAVKNSPFPRSYYRCTTASCNVKKRVERSFRDPSTVVTTYEGQHTH |
| AtWRKY24 | WRKY domain | DDGYRWRKYGQKSVKHNAHPRSYYRCTYHTCNVKKQVQRLAKDPNVVVTTYEGVHNHP |
| AtWRKY25 | WRKY domain-N | NDGYGWRKYGQKQVKKSENPRSYFKCTYPDCVSKKIVETASDGQITEIIYKGGHNHP |
| AtWRKY26 | WRKY domain-N | DDGYNWRKYGQKQVKGSENPRSYFKCTYPNCLTKKKVETSLVKGQMIEIVYKGSHNHP |
| AtWRKY27 | WRKY domain | DLWAWRKYGQKPIKGSPYPRNYYRCSSSKGCLARKQVERSNLDPNIFIVTYTGEHTHP |
| AtWRKY28 | WRKY domain | EDGYRWRKYGQKAVKNSPYPRSYYRCTTQKCNVKKRVERSFQDPTVVITTYEGQHNHP |
| AtWRKY29 | WRKY domain | DAWAWRKYGQKPIKGSPYPRSYYRCSSSKGCLARKQVERNPQNPEKFTITYTNEHNHE |
| AtWRKY30 | WRKY domain | DDGFSWRKYGQKDILGAKFPRGYYRCTYRKSQGCEATKQVQRSDENQMLLEISYRGIHSC |
| AtWRKY31 | WRKY domain | DGCQWRKYGQKMAKGNPCPRAYYRCTMAGGCPVRKQVQRCAEDRSILITTYEGNHNHP |
| AtWRKY32 | WRKY domain-N | DGYNWRKYGQKQVKSPKGSRSYYRCTYTECCAKKIECSNDSGNVVEIVNKGLHTHE |
| AtWRKY33 | WRKY domain-N | EDGYNWRKYGQKQVKGSENPRSYYKCTFPNCPTKKKVERSLEGQITEIVYKGSHNHP |
| AtWRKY34 | WRKY domain-N | DDGYNWRKYGQKLVKGSEYPRSYYKCTHPNCEAKKKVERSREGHIIEIIYTGDHIH |
| AtWRKY35 | WRKY domain | DLWAWRKYGQKPIKGSPYPRGYYRCSSSKGCSARKQVERSRTDPNMLVITYTSEHNHP |
| AtWRKY36 | WRKY domain | NDGCQWRKYGQKTAKTNPLPRAYYRCSMSSNCPVRKQVQRCGEEETSAFMTTYEGNHDHP |
| AtWRKY38 | WRKY domain | DGYLWRKYGQKSIKKSNHQRSYYRCSYNKDHNCEARKHEQKIKDNPPVYRTTYFGHHTCK |
| AtWRKY39 | WRKY domain | DEYSWRKYGQKPIKGSPHPRGYYKCSSVRGCPARKHVERCIDETSMLIVTYEGEHNH |
| AtWRKY40 | WRKY domain | KDGYQWRKYGQKVTRDNPSPRAYFKCACAPSCSVKKKVQRSVEDQSVLVATYEGEHNHP |
| AtWRKY41 | WRKY domain | DDIFSWRKYGQKDILGAKFPRSYYRCTFRNTQYCWATKQVQRSDGDPTIFEVTYRGTHTC |
| AtWRKY42 | WRKY domain | DGCQWRKYGQKMAKGNPCPRAYYRCTMAVGCPVRKQVQRCAEDRTILITTYEGNHNHP |
| AtWRKY43 | WRKY domain | DDGYRWRKYGQKSVKNSLYPRSYYRCTQHMCNVKKQVQRLSKETSIVETTYEGIHNHP |
| AtWRKY44 | WRKY domain-N | DGYNWRKYGQKQVKGSECPRSYYKCTHPKCPVKKKVERSVEGQVSEIVYQGEHNH |
| AtWRKY45 | WRKY domain | DDGYRWRKYGQKAVKNNPFPRSYYKCTEEGCRVKKQVQRQWGDEGVVVTTYQGVHTH |
| AtWRKY46 | WRKY domain | DDGHCWRKYGQKEIHGSKNPRAYYRCTHRFTQDCLAVKQVQKSDTDPSLFEVKYLGNHTCN |
| AtWRKY47 | WRKY domain | NDGCQWRKYGQKMAKGNPCPRAYYRCTMAVGCPVRKQVQRCAEDTTILTTTYEGNHNHP |
| AtWRKY48 | WRKY domain | DDGYRWRKYGQKAVKNSPYPRSYYRCTTVGCGVKKRVERSSDDPSIVMTTYEGQHTHP |
| AtWRKY49 | WRKY domain | DDGYKWRKYGQKSIKNSPNPRSYYKCTNPICNAKKQVERSIDESNTYIITYEGFHFH |
| AtWRKY50 | WRKY domain | DDGFKWRKYGKKMVKNSPHPRNYYKCSVDGCPVKKRVERDRDDPSFVITTYEGSHNH |
| AtWRKY51 | WRKY domain | DDGFKWRKYGKKSVKNNINKRNYYKCSSEGCSVKKRVERDGDDAAYVITTYEGVHNHE |
| AtWRKY52 | WRKY domain | DLWTWRKYGQKDILGSRFPRGYYRCAYKFTHGCKATKQVQRSETDSNMLAITYLSEHNHP |
| AtWRKY53 | WRKY domain | DDVFSWRKYGQKDILGAKFPRSYYRCTHRSTQNCWATKQVQRSDGDATVFEVTYRGTHTC |
| AtWRKY54 | WRKY domain | EDRYAWRKYGQKEILNTTFPRSYFRCTHKPTQGCKATKQVQKQDQDSEMFQITYIGYHTC |
| AtWRKY55 | WRKY domain | DDNHTWRKYGQKEILGSRFPRAYYRCTHQKLYNCPAKKQVQRLNDDPFTFRVTYRGSHTC |
| AtWRKY56 | WRKY domain | DDGYRWRKYGQKSVKNNAHPRSYYRCTYHTCNVKKQVQRLAKDPNVVVTTYEGVHNHP |
| AtWRKY57 | WRKY domain | EDGYRWRKYGQKAVKNSPFPRSYYRCTNSRCTVKKRVERSSDDPSIVITTYEGQHCHQ |
| AtWRKY58 | WRKY domain-N | DDGYNWRKYGQKPIKGCEYPRSYYKCTHVNCPVKKKVERSSDGQITQIIYKGQHDHE |
| AtWRKY59 | WRKY domain | DDGYKWRKYGKKPITGSPFPRHYHKCSSPDCNVKKKIERDTNNPDYILTTYEGRHNHP |
| AtWRKY60 | WRKY domain | KDGYQWRKYGQKITRDNPSPRAYFRCSFSPSCLVKKKVQRSAEDPSFLVATYEGTHNH |
| AtWRKY61 | WRKY domain | NDGCQWRKYGQKIAKGNPCPRAYYRCTIAASCPVRKQVQRCSEDMSILISTYEGTHNHP |
| AtWRKY62 | WRKY domain | DGFLWRKYGQKQIKESEYQRSYYKCAYTKDQNCEAKKQVQKIQHNPPLYSTTYFGQHICQ |
| AtWRKY63 | WRKY domain | DDGFTWRKYGQKTIKTSLYQRCYYRCAYAKDQNCYATKRVQMIQDSPPVYRTTYLGQHTCK |
| AtWRKY64 | WRKY domain | DDGFTWRKYGQKTIKTSPYQRCYYRCTYAKDQNCNARKRVQMIQDNPPVYRTTYLGKHVCK |
| AtWRKY65 | WRKY domain | DSWAWRKYGQKPIKGSPYPRGYYRCSSTKGCPARKQVERSRDDPTMILITYTSEHNHP |
| AtWRKY66 | WRKY domain | DGFIWRKYGQKTIKTSPHQRWYYRCAYAKDQNCDATKRVQKIQDNPPVYRNTYVGQHACE |
| AtWRKY67 | WRKY domain | NDGFTWRKYGQKTIKASAHKRCYYRCTYAKDQNCNATKRVQKIKDNPPVYRTTYLGKHVCK |
| AtWRKY68 | WRKY domain | DDGYKWRKYGQKPVKDSPFPRNYYRCTTTWCDVKKRVERSFSDPSSVITTYEGQHTHP |
| AtWRKY69 | WRKY domain | DSWAWRKYGQKPIKGSPYPRGYYRCSSSKGCPARKQVERSRVDPSKLMITYACDHNHP |
| AtWRKY70 | WRKY domain | EDAFSWRKYGQKEILNAKFPRSYFRCTHKYTQGCKATKQVQKVELEPKMFSITYIGNHTCN |
| AtWRKY71 | WRKY domain | EDGYRWRKYGQKAVKNSPYPRSYYRCTTQKCNVKKRVERSFQDPSIVITTYEGKHNHP |
| AtWRKY72 | WRKY domain | NDGCQWRKYGQKIAKGNPCPRAYYRCTVAPGCPVRKQVQRCADDMSILITTYEGTHSH |
| AtWRKY74 | WRKY domain | DEYSWRKYGQKPIKGSPHPRGYYKCSSVRGCPARKHVERCVEETSMLIVTYEGEHNH |
| AtWRKY75 | WRKY domain | DDGYRWRKYGQKAVKNNKFPRSYYRCTYGGCNVKKQVQRLTVDQEVVVTTYEGVHSHP |
