# Supplementary material for: Genome-wide analysis of the WRKY gene family in the cucumber genome and transcriptome-wide identification of WRKY transcription factors that respond to biotic and abiotic stresses
Source: BMC Plant Biol. 2020 Sep 25;20:443. doi: 10.1186/s12870-020-02625-8 (PMC7517658; doi:10.1186/s12870-020-02625-8)
Supplement: Supplementary file 5 — Additional file 5: Table S3. List of WRKY domain sequences of WRKY IIa proteins. [file 12870_2020_2625_MOESM5_ESM.docx]

**Additional file 5: Table S3** List of WRKY domain sequences of WRKY IIa proteins.

| **Gene ID** | **WRKY domain sequence** |
| --- | --- |
| AtWRKY18 | KDGFQWRKYGQKVTRDNPSPRAYFRCSFAPSCPVKKKVQRSAEDPSLLVATYEGTHNH |
| AtWRKY40 | KDGYQWRKYGQKVTRDNPSPRAYFKCACAPSCSVKKKVQRSVEDQSVLVATYEGEHNHP |
| AtWRKY60 | KDGYQWRKYGQKITRDNPSPRAYFRCSFSPSCLVKKKVQRSAEDPSFLVATYEGTHNH |
| CsWRKY12 | DGYQWRKYGQKVTKDNPSPRAYYKCSFAPTCPVKRKVQRSVEEPCYLVATYEGQHNHP |
| CsWRKY13 | DGYKWRKYGQKITKDNQSPRAYFKCSSPGCPVKKKVQRSLENKSMVIVTYDGHHNHN |
| CsWRKY38 | DGYQWRKYGQKVTRDNPSPRAYFKCSSAPNCPVKKKVQRSLEDPTILVATYEGEHSH |
| VvWRKY3-2 | RVVVRTEKSDNSLLLKDGYQWRKYGQKVTRDNPSPRAYFKCSFAPTCPVKKKVQRSAEDQSLLIATYEGEHNHQQP |
| VvWRKY4 | RVYTRTDASDTSLVVKDGYQWRKYGQKVTRDNPSPRAYFKCSFAPSCPVKKKVQRSVEDQSILVATYEGEHNHPHP |
| VvWRKY18 | QVFVRTNPKDKSLVMKDGFQWRKYGQKITKDNPSPRAYFRCSMAPQCPVKKKVQRCMEDSSILVATYEGAHNHEPP |
| VvWRKY40-1 | RVYTRTDASDTSLVVKDGYQWRKYGQKVTRDNPSPRAYFKCSFAPSCPVKKKVQRSVEDQSILVATYEGEHNHPHP |
| VvWRKY40-2 | RVVVRTEKSDNSLLLKDGYQWRKYGQKVTRDNPSPRAYFKCSFAPTCPVKKKVQRSAEDQSLLIATYEGEHNHQQP |
| PtrWRKY18 | VRDGYQWRKYGQKVTRDNPSPRAYFKCSFAPSCPVKKKVQKSAENPSILVATYEGEHNH |
| PtrWRKY35 | VKDGYQWRKYGQKVTRDNPSPRAYYKCSFAPSCPVKKKVQRSAEDPSILVATYEGEHNH |
| PtrWRKY40 | VKDGYQWRKYGQKVTRDNPSPRAYFKCSFAPSCPVKKKVQRSIDDQSVLVATYEGEHNH |
| PtrWRKY60 | VKDGYQWRKYGQKVTRDTRAYFKCSFAPSCPVKKKVQRSIDDQSVLVATYEGEHNH |
| PtrWRKY77 | VKDGYQWRKYGQKVTRDNPSPRAYFRCSMAPNCPVKKKVQRCVEDDSVLVASYDGEHNH |
| SlWRKY40 | DTSLIVKDGYQWRKYGQKVTRDNPCPRAYFRCSFAPGCPVKKKVQRSIEDQSIVVATYEGEHNHPQT |
| SlWRKY39 | DTSLIVKDGYQWRKYGQKVTRDNPSPRAYFKCSFAPTCPVKKKVQRSVEDQSILVATYEGEHNHSKV |
| SlWRKY43 | HTTLLVKDGYEWRKYGQKVTRNNPYPRAYFKCSFAPTCPVKKKVQRSIDDMSIIIATYEGEHNHSKV |
| SlWRKY45 | DQTSVVKDGYNWRKYGQKVTRDNPYPRAYYKCSFAPTCPVKKKVQRSIEDPSILVGVYEGEHNHPHP |
| SlWRKY46 | DQTSVVKDGYNWRKYGQKVTRDNPSPRAYFKCSFAPSCPVKKKVQRSVKDASILVATYEGEHNHPQP |
| OsWRKY62 | VKDGYQWRKYGQKVTRDNPYPRAYFRCAFAPSCPVKKKLQRCAEDRSMLVATYEGEHNHAL |
| OsWRKY28 | VKDGYQWRKYGQKVTKDNPCPRAYFRCSFAPACPVKKKVQRSADDNTVLVATYEGEHNHAQ |
| OsWRKY71 | VKDGYQWRKYGQKVTKDNPCPRAYFRCSFAPACPVKKKVQRSAEDNTILVATYEGEHNH |
| OsWRKY76 | VKDGYQWRKYGQKVTRDNPSPRAYFRCAFAPSCPVKKKVQRSAEDSSLLVATYEGEHNHPH |
| ZmWRKY21 | VKDGYQWRKYGQKVTRDNPSPRAYFRCAYGPSCPVKKKVQRSAEDSSVLVATYEGEHNHPC |
| ZmWRKY62 | VKDGYQWRKYGQKVTKDNPCPRAYFRCSFAPACPVKKKVQRSADDPTILVATYEGDHNHGQ |
| ZmWRKY67 | VKDGYQWRKYGQKVTKDNPCPRAYFRCSLAPSCPVKKKVQRSADDSAVLVATYEGEHNHAR |
| ZmWRKY80 | VKDGYQWRKYGQKVTRDNPSPRAYFRCAYAPSCPVKKKVQRSAEDSAMLVATYEGEHNHPS |
| ZmWRKY81 | LRDCYQWRKYGQKVTRDNPYPRSYFRCAYAPSCPVKKKVQRSADDNLMLVATYEGEHNHEQ |
| ZmWRKY109 | VKDGYQWRKYGQKVTKDNPCPRAYFRCSFAPSCPVKKKVQRSADDRTILVATYEGDHNHAQ |
| TaWRKY4 | HADPADLSLVVKDGYQWRKYGQKVTKDNPCPRAYFRCSFAPGCPVKKKVQRSAEDKTILVATYEGEHNHSQP |
| TaWRKY8-A | HADPADLSLVVKDGYQWRKYGQKVTKDNPCPRAYFRCSFAPGCPVKKKVQRSAEDKTILVATYEGEHNHSQP |
| TaWRKY34 | HADPSDLSLVVKDGYQWRKYGQKVTKDNPC |
| TaWRKY39 | HADPADLSLVVKDGYQWRKYGQEGDQGQPMPEGLLPVLRSRRPAR |
| TaWRKY43 | HADPSDLSLVVKDGYXWRKYGQKVTQEQPFALRATSGC |
| TaWRKY52 | RIDPSDTSLVVKDGYQWRKYGQKVTRDNPSPRAYFRCAFAPSCPVKKKVQRSAEDSSVVEATYEGEHNHPRP |
| TaWRKY52-A | RIDPSDTSLVVKDGYQWRKYGQKVTRDNPSPRAYFRCAFAPSCRVKKKVQRSAEDSSVVEATYEGEHNHPHP |
| TaWRKY54 | HADPADLSLVVKDGYQWRKYGQKVTKGHPCPRAYFRCSFRPGCPVKKKVQRSAQDKTILVGTYEGENTTASP |
| TaWRKY60 | RVRPSDLSLVVKDGHQWLKYGQKVTKDNPCPRAYFRCSFAPSCQVKKKVQRSAEDKAVLVATYDGDHNHAQP |
| TaWRKY71 | HADPADLSLVLKDGYQWRKYGQKVTKDNPCPRAYFRCSFAPGCPVKKKVQRSAEDKTILVATYEGEHNHSQP |
| TaWRKY79 | HADPSDLSLVVKDGYQWRKYGQKVTKDNPCPRAYFRCSFAPSCQVKKKVQRSAEDKAVILVATYEGEHNHAQP |
| TaWRKY79-A | HADPADLSLVVKDGYQWRKYGKKVTKDNPCPRAYFRCSFAPSCQVKKKVQRSAQDKNVLVATYEGEHNTHSP |
| TaWRKY80 | HADPSDLSLVVKDGYQWRKYGQKVTKDNPCPRAYFRCSFAPGCPVKKKVXEERQDKTILVATYEGEHNHTQP |
| TaWRKY80-A | HADPSDLSLVVKDGYQWRKYGQKVTXDNPCPRAYFRCSFAPGCPVKKKVQRSAEDKNILVATYEGEHNTPSP |
| TaWRKY80-B | HADPADLSLVLKDGYQWRKYGQKVTKDNPCPRAYFRCSFAPGCPVKKKVQRSAEDKTILVATYEGEHNHSQP |
| TaWRKY81 | RRPPEPQPXVLRDRYQLRKYGPKVTKANPCPRAYFGWSLAPGCPVKKKVQRSPEDKTILVATYEGEHNHSQP |
| StWRKY015 | MNDGCQWRKYGQKIAKGNPCPRAYYRCTVAPNCPVRKQVQRCIQDMSILITTYEGTHNHPL |
| StWRKY016 | INDGCQWRKYGQKISRGNPCPRSYYRCSVAPLCPVRKQVQRCLEDMSILITTYEGTHNHS |
| StWRKY017 | MNDGCQWRKYGQKIAKGSPNCPRAYYRCTVAPGCPVRKQVQRCLEDMSILITTYEGTHNH |
| StWRKY018 | ITDGCQWRKYGQKMAKGNPCPRAYYRCTMAAGCPVRKQVQRCAEDRTILNTTYEGTHNHPL |
| StWRKY019 | ISDGCQWRKYGQKMAKGNPCPRAYYRCTMAAGCPVRKQVQRCADDRTILITTYEGAHNHPL |
| RcWRKY10 | LIVKDGYQWRKYGQKVTRDNPSPRAYFKCSFAPSCPVKKKVQRSIEDQTILVATYEGEHNH |
| RcWRKY12 | LVVKDGYQWRKYGQKVTRDNPSPRAYYKCSFAPSCPVRKKKL |
| RcWRKY11 | GMADDGYKWRKYGQKSIKNSPFPRSYYRCTNPRCSAKKQVERSSEDQDTLVITYEGLHLH |
| Pbr019030 | LIVNDGYQWRKYGQKVTRDNPCPRAYYKCSFAPSCPVKKKVQKSAENPCLLVATYEGEHNH |
| Pbr020001 | LIVKDGYQWRKYGQKVTRDNPSPRAYYKCSFAPSCPVKKKVQKSAENPCVLVATYEGEHNH |
| Pbr019026 | LIVKDGYQWRKYGQKVTKDNPSSPRAYFRCSFAPRCPVKKKVQRCIEDDSILVATYEGEHNH |
| Pbr020000 | LIVKDGYQWRKYGQKVTKDNPSSPRAYFRCSFAPRCPVKKKVQRSMEDNSFLMVTYEGEHNH |
| Pbr022408 | LVVKDGYQWRKYGQKVTRDNPCPRAYFKCSFAPSCPVKKKVQRSVEDQTILVGTYEGEHNH |
| Pbr004885 | LVVKDGYQWRKYGQKVTRDNPCPRAYFKCSFAPSCPVKKKVQRSVEDQSILVATYEGEHNH |
| HvWRKY3 | DTSLVVKDGYQWRKYGQKV-TRDNPSPRAYFRCAF--APSCPVKKKVQRSAE-DSSMVEATYEG-EHNHPRP |
| HvWRKY1 | DLSLVVKDGYQWRKYGQKV-TKDNPCPRAYFRCSF--APGCPVKKKVQRSAE-DKTILVATYEG-EHNHTQP |
| HvWRKY2 | DLSLVVKDGYQWRKYGQKV-TKDNPCPRAYFRCSS--APSCQVKKKVQRSAE-DKTVLVATYDG-DHNHAPP |
| HvWRKY23 | DLSLVVKDGYQWRKYGQKV-TKDNPCPRAYFRCSF--APSCQVKKKVQRSAE-DKTVLVATYDG-DHNHAPP |
| HdWRKY14 | LVVKDGYQWRKYGQKVTRDNPSPRAYFRCAFAPSCPIKKKVQRSAENSSVLEATYEGEHNHPQ |
| HdWRKY39 | LVVKDGYQWRKYGQKVTKDNPCPRAYFRCSFAPACPVKKKVQRSAEDRTVLVATYEGEHNHGQ |
| HdWRKY68 | LVVKDGYQWRKYGQKVTKDNPCPRAYFRCSFAPACPVKKKVQRSADDKALLVATYEGDHNHAQ |
| SiWRKY003 | VKDGYQWRKYGQKVTKDNPCPRAYFRCSFAPACKVKKKVQRSADDNTILVATYEGCHNH |
| SiWRKY014 | VKDGYQWRKYGQKVTRDNPSPRAYFRCAFAPSCKVKKKVQRSAEDSSMLVATYEGCHNH |
| SiWRKY015 | LKDGYQWRKYGQKVTRDNPYPRAYFRCAYAPSCKVKKKVQRSAEDKSMLVATYEGKHNH |
| SiWRKY042 | VKDGYQWRKYGQKVTKDNPCPRAYFRCSFAPACKVKKKVQRSAEDKTILVATYEGAHNH |
| SvWRKY041 | VKDGYQWRKYGQKVTKDNPCPRAYFRCSFAPACRVKKKVQRSAEDKTILVATYEGAHNH |
