# Supplementary material for: Genome-wide analysis of the WRKY gene family in the cucumber genome and transcriptome-wide identification of WRKY transcription factors that respond to biotic and abiotic stresses
Source: BMC Plant Biol. 2020 Sep 25;20:443. doi: 10.1186/s12870-020-02625-8 (PMC7517658; doi:10.1186/s12870-020-02625-8)
Supplement: Supplementary file 11 — Additional file 11: Figure S3. Hierarchical clustering of cucumber gene expression profiles under heat treatment. A, Hierarchical cluster of expressed cucumber genes in 9 samples. In the color panels, each transverse line represents a single gene and the color of the line indicates the expression level of the gene relative to the mean center in a specific sample: red, high expression; green, low expression level. B, The hierarchical clustering on the gene expression matrix, using the Pearson correlation coefficient as a proxy of similarity between transcriptomes. HT0h = heat treatment for 0 h (hours), HT3h = heat treatment for 3 h, HT6h = heat treatment for 6 h. [file 12870_2020_2625_MOESM11_ESM.pptx]

## Slide 1
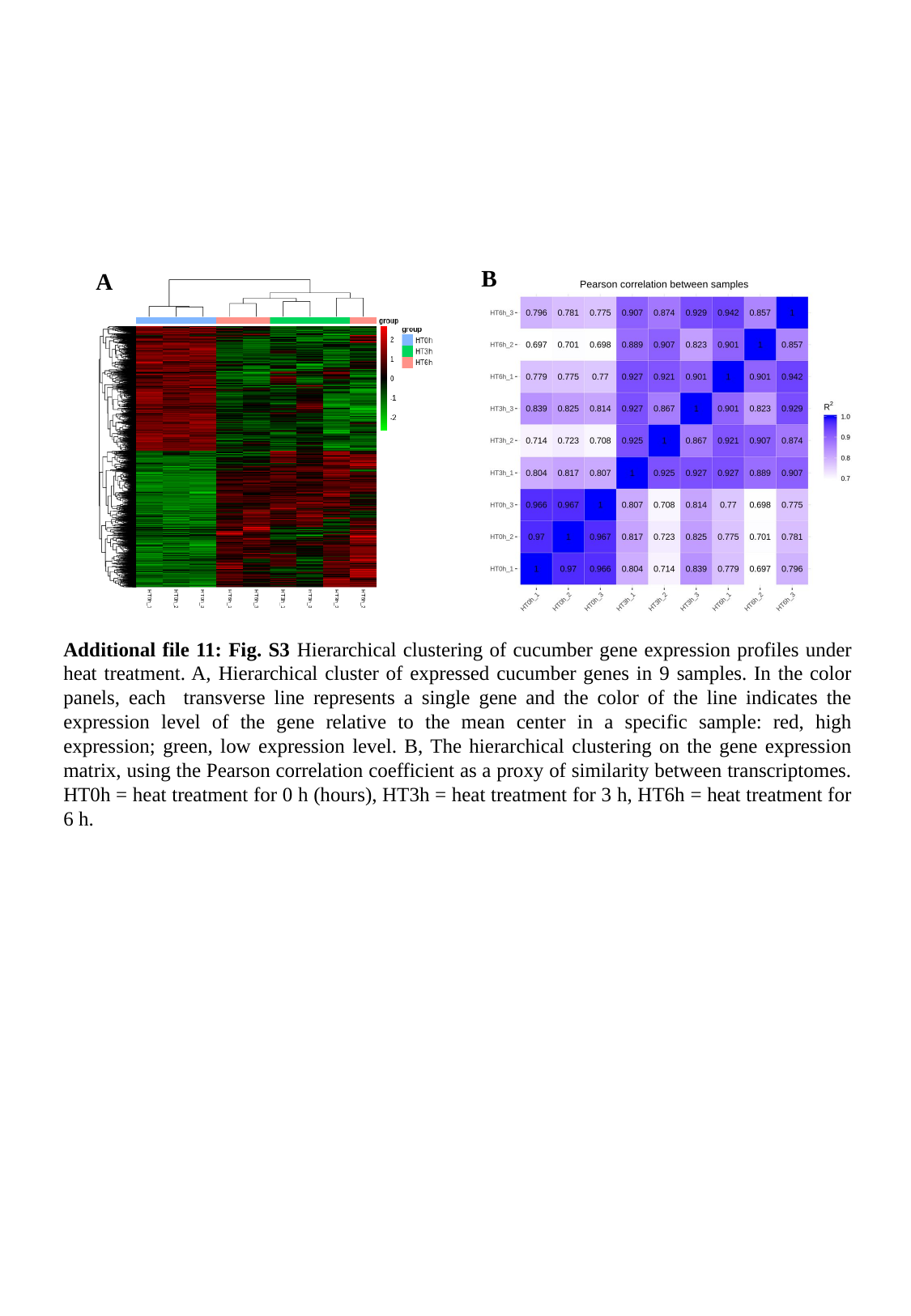

B
A
Additional file 11: Fig. S3 Hierarchical clustering of cucumber gene expression profiles under heat treatment. A, Hierarchical cluster of expressed cucumber genes in 9 samples. In the color panels, each transverse line represents a single gene and the color of the line indicates the expression level of the gene relative to the mean center in a specific sample: red, high expression; green, low expression level. B, The hierarchical clustering on the gene expression matrix, using the Pearson correlation coefficient as a proxy of similarity between transcriptomes. HT0h = heat treatment for 0 h (hours), HT3h = heat treatment for 3 h, HT6h = heat treatment for 6 h.
